# Bidirectional Causal Associations Between Inflammatory Bowel Disease and Ankylosing Spondylitis: A Two-Sample Mendelian Randomization Analysis

**DOI:** 10.3389/fgene.2020.587876

**Published:** 2020-11-19

**Authors:** Zhiyong Cui, Guojin Hou, Xiangyu Meng, Hui Feng, Baichuan He, Yun Tian

**Affiliations:** ^1^Department of Orthopedic Surgery, Peking University Third Hospital, Beijing, China; ^2^Peking University Health Science Center, Beijing, China; ^3^Department of Urology, Zhongnan Hospital of Wuhan University, Wuhan, China

**Keywords:** ankylosing spondylitis, inflammatory bowel disease, ulcerative colitis, Crohn’s disease, Mendelian randomization

## Abstract

**Background:**

Associations between inflammatory bowel disease (IBD) [including ulcerative colitis (UC) and Crohn’s disease (CD)] and ankylosing spondylitis (AS) were discovered in observational studies, but no evidence supported the causal relationship between the two diseases.

**Methods:**

We employed two-sample Mendelian randomization (MR) to estimate the unconfounded bidirectional causal associations between IBD (including UC and CD) and AS. We selected single-nucleotide polymorphisms (SNPs) from genome-wide association studies (GWAS) after strictly assessing the quality of the studies in the IEU GWAS database. Sensitivity analyses were also conducted to verify whether heterogeneity and pleiotropy can bias the MR results.

**Results:**

We found positive causal effects of genetically increased UC, CD, and IBD risk on AS (e.g., UC and AS, IVW OR: 1.0256, 95% CI: 1.0130∼1.0385, *p* = 6.43E-05). However, we did not find significant causal associations of AS with UC, CD, or IBD (e.g., AS and UC, IVW OR: 1.1858, 95% CI: 0.8639∼1.6278, *p* = 0.2916). The sensitivity analysis also confirmed that horizontal pleiotropy was unlikely to bias the causality (e.g., UC and AS, MR-Egger: intercept *p* = 0.1326). The leave-one-out analysis also demonstrated that the observed links were not driven by SNP. No evidence of heterogeneity was found between the genetic variants (e.g., UC and AS, MR-Egger: *Q* statistic = 43.1297, *I*^2^<0.0001, *p* = 0.7434).

**Conclusion:**

Our results provide new evidence indicating there are positive causal effects of IBD on AS in the European population. We suggest that the features of inflammatory bowel disease in particular should be assessed in the diagnosis of ankylosing spondylitis. We also provide some advice for preventing and treating the two diseases.

## Introduction

Ankylosing spondylitis (AS) is a type of immune-mediated inflammatory rheumatic and spinal disease in the axial spondyloarthritis (SpA) spectrum, and it is also termed radiographic axial SpA (Raychaudhuri and Deodhar, 2014; Sieper and Poddubnyy, 2017). Ulcerative colitis (UC) and Crohn’s disease (CD) are two main forms of inflammatory bowel disease (IBD) (Abraham and Cho, 2009; Ordás et al., 2012; Torres et al., 2012). It has been suggested that IBD results from an inappropriate inflammatory response to intestinal microbes in a genetically susceptible host.

It has long been recognized that there is a close relationship between IBD and AS. Patients with IBD frequently suffer from extraintestinal symptoms, the most common symptoms of which are musculoskeletal manifestations (Larsen et al., 2010). It has been estimated that the prevalence of AS in IBD patients is approximately 3%, as reported by a meta-analysis (Karreman et al., 2016). Because both diseases likely occur concomitantly, some researchers suggest that AS and IBD might share a similar pathogenesis, but there is no evidence showing that the two conditions have a causal relationship. Exploring the causal relationship between the diseases is of great significance and may increase the current knowledge of the pathogeneses of AS and IBD and improve treatments.

Observational studies conducted to estimate causal inference have numerous inherent limitations, such as remaining limited to known and properly measured confounders (Greenland and Morgenstern, 2001). Therefore, we used Mendelian randomization (MR), which uses instrumental variables (IVs) in the analysis of genetic variants, to determine whether an observational association between exposures and outcomes exists and is consistent with a causal effect. MR rests on three assumptions: (a) the genetic variant is associated with the exposures; (b) the genetic variant is not associated with confounders; and (c) the genetic variant influences the outcomes only through the exposures. The genetic variants used in MR are available due to genome-wide association studies (GWAS) being conducted and high-throughput genomic technologies being developed. In this study, we used single-nucleotide polymorphisms (SNPs) strongly associated with IBD (including UC and CD) and AS as IVs. We performed two-sample MR using the effects of IVs on the exposures (UC, CD, and IBD) and outcomes (AS) from two independent samples. We analyzed the summary-level data and used statistical methods to obtain quantitative estimates of the effects of UC, CD, and IBD on AS. Moreover, we also used reverse MR to investigate the bidirectional causal relationship between IBD and AS.

## Materials and Methods

### Data Source

In our study, a crucial step in performing MR was to choose appropriate genetic variants from the publicly available GWAS database to serve as valid IVs for IBD. We selected SNPs as IVs for all exposures (UC, CD, and IBD) and outcomes (AS) from the IEU GWAS database, a database of genetic associations from GWAS summary datasets^1^ (Hemani et al., 2018). SNPs associated with IBD were derived from a transancestry association study on IBD, which was performed by the International Inflammatory Bowel Disease Genetics Consortium (IIBDGC) (Liu et al., 2015). In the association study, IBD was diagnosed on the basis of the accepted radiological, endoscopic, and histopathological evaluations. The summary-level data on the impact of the IBD-associated SNPs on AS were derived from GWAS, which were performed by the International Genetics of Ankylosing Spondylitis Consortium (IGAS) (International Genetics of Ankylosing Spondylitis Consortium[IGAS] et al., 2013). In the GWAS, AS was diagnosed on the basis of the modified New York Classification Criteria (van der Linden et al., 1984). Population stratification is a potential source of bias for MR analyses. Because there are differences in allele frequencies, one SNP can be associated with ancestry, which itself can be associated with disease risk (Emdin et al., 2017; Larsson et al., 2019). To prevent population stratification bias, we selected SNPs and their corresponding summary statistics (*p*-value, beta effect, and standard error) from studies that included only individuals of European descent for both the exposures and outcomes.

### SNP Selection

From the GWAS summary results, we conducted a series of quality control steps to select eligible SNPs. We selected SNPs with a genome-wide association (*p* < 5E−08), with independent inheritance (*r*^2^ < 0.01), and without linkage disequilibrium (LD) in summary statistics. When the target SNPs were not available in the outcomes of a study, we used proxy SNPs that had high LD (*r*^2^ > 0.8) with the SNPs of interest. We selected the reference sample derived from European ancestral individuals from the 1,000 Genomes Project to estimate the allele frequency and LD level^2^ (1000 Genomes Project Consortium et al., 2010). The palindromic SNPs with intermediate allele frequencies (palindromic SNPs referred to the SNPs with A/T or G/C alleles and “intermediate allele frequencies” referred to 0.01 < allele frequency < 0.30) were excluded from the above selected instrument SNPs. SNPs with a minor allele frequency (MAF) of < 0.01 were also excluded. We also calculated the F statistics for the SNPs to measure the strength of the instruments. IVs with an F statistic less than 10 were excluded and were often labeled as “weak instruments” (Burgess et al., 2015). These rigorously selected SNPs were used as the final instrumental SNPs for the subsequent MR analysis. The proportion of phenotypic variation explained by IV SNPs was also estimated.

### Effect Size Estimate

We applied the principles of two-sample MR to assess the role of exposures (UC, CD, and IBD) in the susceptibility of the outcomes (AS). We chose the SNPs according to the selection criteria listed above as our instrumental variables and estimated the effects of the selected SNPs on the exposures and outcomes. We verified the stability of the results by comparing the effect directions across different two-sample MR filtering methods (Emdin et al., 2017; Larsson et al., 2019). The causal associations between exposures (UC, CD, and IBD) and outcomes (AS) were estimated with inverse variance weighted (IVW), MR-Egger, and the weighted median (WM). The IVW method uses a meta-analysis approach to combine the Wald ratios of the causal effects of each SNP and can provide the most precise estimates. The WM estimate provides a reliable effect estimate of the causal effect when at least 50% of the weight in the analysis comes from effective IVs. MR-Egger regression is used to create a weighted linear regression of the outcome coefficients with the exposure coefficients. The WM method offers some important advantages over MR-Egger because it has improved precision and is more robust to violations in the causal effects. MR-Egger estimates may be inaccurate and can be strongly influenced by outlying genetic variants (Bowden et al., 2016).

We also performed a recently developed method called the Robust Adjusted Profile Score (MR.RAPS) to estimate the causal effects, which can lead to a considerably higher statistical power than the conventional MR analysis can, which only uses a small set of strong instruments (Zhao et al., 2019). MR.RAPS considers the measurement error in SNP-exposure effects and is unbiased when there are many weak instruments, and is robust to systematic and idiosyncratic pleiotropy (Zhao et al., 2019). The MR.RAPS method can alleviate but cannot solve the problem of horizontal pleiotropy (Zhao et al., 2019).

### Sensitivity Analyses

To exclude possible violations of the MR assumptions, we conducted multiple sensitivity analyses to verify whether heterogeneity and pleiotropy within the genetic instruments tested can bias the MR results. Pleiotropy refers to the phenomenon in which a single locus affects multiple phenotypes. Horizontal pleiotropy arises when a genetic variant is associated with more than one phenotype on separate pathways, which can invalidate the results from MR analyses. We performed MR-Egger regression to assess and adjust for horizontal pleiotropy, as it is a method that can identify confounders that may distort the MR results. We evaluated the MR-Egger regression intercept and conducted the MR-PRESSO (Pleiotropy RESidual Sum and Outlier) global test (Verbanck et al., 2018) to estimate the presence of pleiotropy. MR-PRESSO is an extension of previous approaches that utilize the general model of multi-instrument MR on summary statistics and is best used to identify inconsistencies between genetic associations of different genetic variants and remove outlying genetic variants (Verbanck et al., 2018). In addition, to test for the presence of pleiotropy, we evaluated the pleiotropic effects of UC, CD, and IBD on osteoarthritis (OA), as these effects might distort the effects of UC, CD, and IBD on AS. Summary statistics for OA were extracted from studies performed by the GWAS of European descent performed by Arthritis Research UK Osteoarthritis Genetics (arcOGEN) Consortium (arcOGEN Consortium et al., 2012). We assessed the potential associations between the SNPs that were extracted for the MR analysis and OA. Variants with detectable associations with OA were removed from the MR analysis, and the remaining non-pleiotropic variants were taken as instruments for the MR analysis. Associations of the SNPs with OA were considered statistically significant at a Bonferroni-corrected *p* < 0.05/N, with N representing the number of SNPs in each exposure trait.

We used the IVW, WM, and maximum likelihood methods to evaluate the heterogeneity among SNPs. The level of heterogeneity was quantified by Cochran Q statistics and *I*^2^ statistics. The Cochran Q statistic was calculated as the weighted sum of the squared differences between individual SNP effects and the pooled effect across all SNPs.

An *I*^2^ statistic calculation adapted for meta-analyses was used to quantify the strength of the violation for MR-Egger. The values are between 0 and 1 and indicate the expected relative bias of the MR-Egger causal estimate in the two-sample MR context (Bowden et al., 2016). Moreover, the causal directions between the exposures and outcomes were tested by the MR-Steiger method (Hemani et al., 2017). To guarantee that the MR estimates are not influenced by the inclusion of proxy SNPs, we implemented a “leave-one-out” sensitivity analysis by removing a different SNP in each iteration when performing the MR. All statistical tests were two-sided, and the results of the MR analyses and sensitivity analyses regarding the causal effects of UC, CD, and IBD on AS were considered statistically significant at *p <* 0.05.

### Bidirectional Mendelian Randomization

We also sought to explore whether AS influenced UC, CD, and IBD. Therefore, we reversed the functions of the exposures and outcomes to perform a bidirectional MR analysis and determine the effects of a genetically increased risk of AS on UC, CD, and IBD. To that end, we selected SNPs that were significant genome-wide (*p* < 5E−08) and independently inherited (*r^2^* < 0.01) without LD for AS from IGAS (International Genetics of Ankylosing Spondylitis Consortium[IGAS] et al., 2013). We then used the corresponding effect estimates from IIBDGC as the outcomes (Liu et al., 2015). We then applied the same MR methods as above. The statistical tests of the bidirectional MR analysis were two-sided, and the results of the MR analyses and sensitivity analyses regarding the causal effects of AS on UC, CD, and IBD were considered statistically significant at a Bonferroni-corrected *p <* 0.0167 (e.g., 0.05/3 outcomes).

All statistical tests were performed using the “TwoSampleMR” package for R language, version 3.6.1 (R Foundation for Statistical Computing, Vienna, Austria). The “TwoSampleMR” codes in our study were available here: https://mrcieu.github.io/TwoSampleMR.

## Results

We incorporated 52, 59, and 82 significant (*p* < 5E−08) and independent SNPs (*r^2^* < 0.01) as IV SNPs for UC, CD, and IBD, respectively. However, three SNPs (rs3135501, rs11641016, and rs2564117) for IBD that were palindromic with intermediate allele frequencies were excluded. Finally, a total of 52, 59, and 79 IVs of UC, CD, and IBD were carefully selected (Table 1). Overall, the selected instruments explained approximately 21.49, 28.94, and 21.69% of the phenotypic variation in UC, CD, and IBD, respectively, on the observed scale. For these instrumental variables, all the *F*-values were larger than 10 (ranging from 29.7576 to 110.7637 for UC; ranging from 30.7373 to 349.9869 for CD and ranging from 30.9495 to 232.7940 for IBD) with average *F*-values of 45.7381, 66.9439, and 52.0463 for UC, CD, and IBD, respectively; these results indicate that the variables satisfy the strong relevance assumption of MR and that the instrument bias is weak and cannot substantially influence the estimations of causal effects (Supplementary Tables S1–S3).

**TABLE 1:** MR estimates from each method of assessing the causal effects of ulcerative colitis, Crohn’s disease, and IBD on ankylosing spondylitis risk.

Exposure traits	MR methods	Ankylosing spondylitis				
		Number of SNPs	OR (95% CI)	SE	MR *p-*value	MR-Steiger test
Ulcerative colitis	MR-Egger	52	0.9927 (0.9502∼1.0370)	0.0223	0.7426	Direction: TRUE *p*-value <0.0001
	Inverse variance weighted	52	1.0256 (1.0130∼1.0385)	0.0063	*6.43E-05*	
	Weighted median	52	1.0241 (1.0054∼1.0432)	0.0094	*0.0115*	
	MR.RAPS	52	1.0280 (1.0146∼1.0414)	0.0067	*3.59E-05*	
	MR-PRESSO test	52	1.0256 (1.0137∼1.0377)	0.0060	*9.63E-05*	

Crohn’s disease	MR-Egger	59	1.0015 (0.9702∼1.0337)	0.0162	0.9282	Direction: TRUE *p*-value <0.0001
	Inverse variance weighted	59	1.0194 (1.0088∼1.0302)	0.0054	*0.0003*	
	Weighted median	59	1.0235 (1.0069∼1.0404)	0.0083	*0.0054*	
	MR.RAPS	59	1.0214 (1.0103∼1.0327)	0.0056	*0.0002*	
	MR-PRESSO test	59	1.0194 (1.0096∼1.0293)	0.0049	*0.0002*	

IBD	MR-Egger	79	0.9920 (0.9512∼1.0345)	0.0214	0.7078	Direction: TRUE *p*-value <0.0001
	Inverse variance weighted	79	1.0259 (1.0133∼1.0387)	0.0063	*5.25E-05*	
	Weighted median	79	1.0352 (1.0153∼1.0556)	0.0099	*0.0005*	
	MR.RAPS	79	1.0292 (1.0160∼1.0427)	0.0066	*1.33E-05*	
	MR-PRESSO test	79	1.0238 (1.0123∼1.0355)	0.0058	*0.0001*	

*MR, Mendelian randomization; RAPS, Robust Adjusted Profile Score; PRESSO, Pleiotropy RESidual Sum and Outlier; SNP, single nucleotide polymorphism; IBD, inflammatory bowel disease; OR, odds ratio; CI, confidence interval; SE, standard error (the standard error is an estimate of the standard deviation (SD) of the coefficient). The italic values mean the statistical significance.*

**FIGURE 1:**
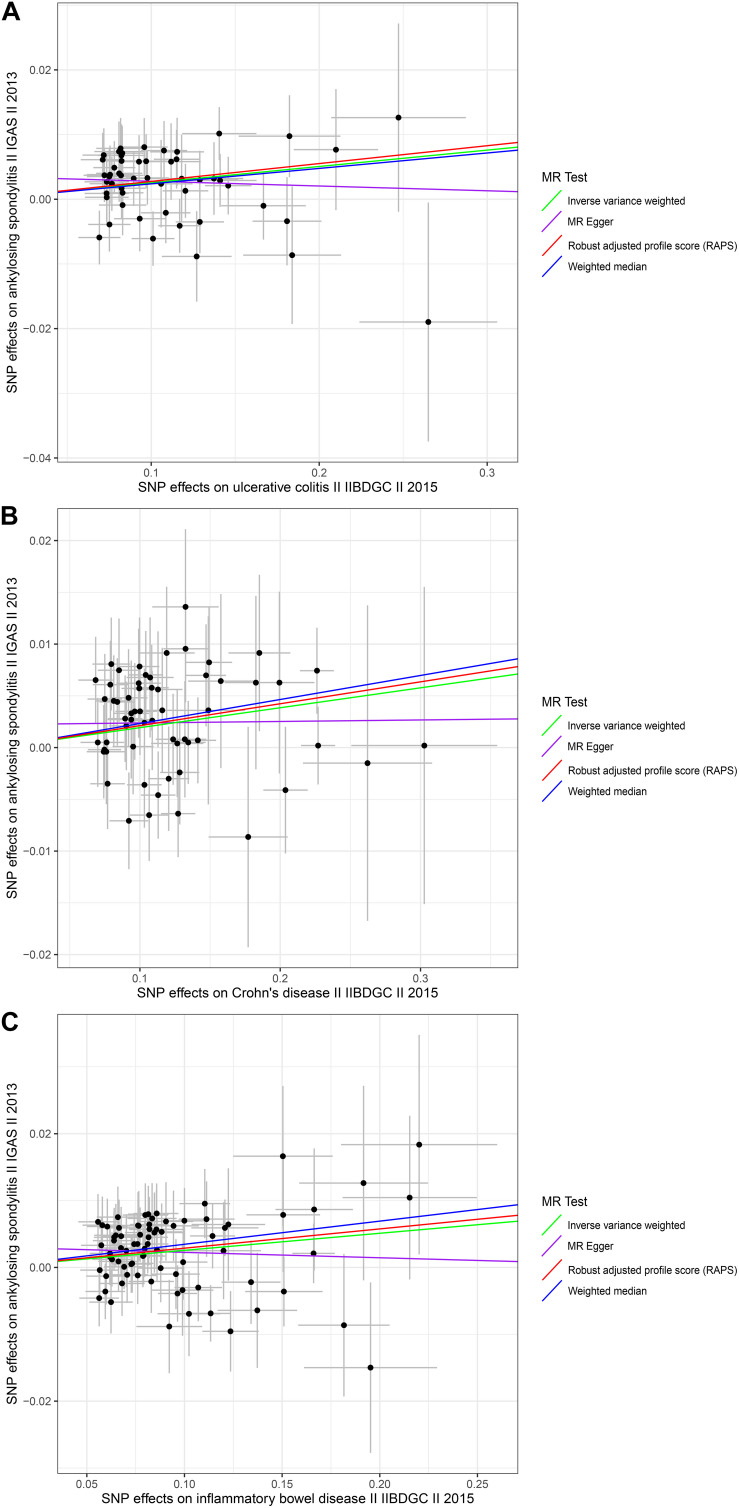
Scatter plots of the genetic associations with inflammatory bowel disease against ankylosing spondylitis risk using different MR methods. **(A)** Ulcerative colitis against ankylosing spondylitis risk; **(B)** Crohn’s disease against ankylosing spondylitis risk; and **(C)** inflammatory bowel disease against ankylosing spondylitis risk. The slopes of each line represent the causal association for each method. The green line represents the inverse variance weighted estimate, the purple line represents the MR-Egger estimate, the red line represents the MR.RAPS estimate, and the blue line represents the weighted median estimate.

The causal associations between UC and AS determined using the full set of 52 SNPs were not consistent among the three MR methods. The IVW and WM MR results showed that the per unit increase in the log-odds of having UC was significantly associated with an increased risk of having AS at *p* < 0.05 (IVW OR = 1.0256, 95% CI 1.0130–1.0385, *p* = 6.43E-05; and WM OR = 1.0241, 95% CI 1.0054–1.0432, *p* = 0.0115), while the MR-Egger regression method did not suggest a significant association between CD and AS (OR = 0.9927, 95% CI 0.9502–1.0370, *p* = 0.7426) (Table 1 and Figure 1). Given that the IVW estimates were consistent with the WM estimates and that the IVW estimates may be unbiased estimates of causal effects and are considerably more powerful than the MR-Egger regression estimates (Bowden et al., 2016), we believe that UC had a positive causal effect on AS risk. The causal effects of CD and IBD on AS were the same as those of UC at *p* < 0.05 (For CD, IVW OR = 1.0194, 95% CI 1.0088–1.0302, *p* = 0.0003; WM OR = 1.0235, 95% CI 1.0069–1.0404, *p* = 0.0054 and MR-Egger OR = 1.0015, 95% CI 0.9702–1.0337, *p* = 0.9282. For IBD, IVW OR = 1.0259, 95% CI 1.0133–1.0387, *p* = 5.25E-05; WM OR = 1.0352, 95% CI 1.0153–1.0556, *p* = 0.0005 and MR-Egger OR = 0.9920, 95% CI 0.9512–1.0345, *p* = 0.7078) (Table 1 and Figure 1). Moreover, the MR.RAPS results were found to be consistent with the MR IVW and WM results, showing that UC, CD, and IBD were significantly associated with an increased risk of having AS at *p* < 0.05 (For UC, MR.RAPS OR = 1.0280, 95% CI 1.0146–1.0414, *p* = 3.59E-05. For CD, the MR.RAPS results were as follows: OR = 1.0214, 95% CI 1.0103–1.0327, *p* = 0.0002. For IBD, the MR.RAPS results were as follows: OR = 1.0292, 95% CI 1.0160–1.0427, *p* = 1.33E-05) (Table 1 and Figure 1). Therefore, we found positive causal associations of UC, CD, and IBD with an increased risk of AS with the MR IVW, WM, MR-Egger, and MR.RAPS methods.

We conducted MR-Egger regression to assess pleiotropy, and the results revealed that horizontal pleiotropy was unlikely to bias the causality of UC (*p* = 0.1326), CD (*p* = 0.2484), and IBD (*p* = 0.1044) with AS (Table 2). The “leave-one-out” analysis also revealed that no single SNP was driving the MR estimates (see Supplementary Figures S1–S3). The associations between these genetic variants and confounding factors OA were also analyzed. None of the genetic variants of the UC, CD, or IBD traits were significantly associated with OA at the Bonferroni-corrected significance threshold of *p* < 0.0010 (e.g., 0.05/52), *p* < 0.0008 (e.g., 0.05/59), or *p* < 0.0006 (e.g., 0.05/79) (Supplementary Tables S4–S6). Cochran *Q*-value and the *I*^2^-value indicated there was no heterogeneity between the IV estimates determined with the IVW, MR-Egger, and maximum likelihood methods (For UC, MR-Egger Q = 43.1297, *I^2^* < 0.0001, *p* = 0.7434; IVW Q = 45.4670, *I*^2^ < 0.0001, *p* = 0.6923; maximum likelihood Q = 45.2706, *I*^2^ < 0.0001, *p* = 0.6996. For CD, the MR-Egger results were as follows: Q = 47.4296, *I*^2^ < 0.0001, *p* = 0.8129; IVW Q = 48.7894, *I*^2^ < 0.0001, *p* = 0.8002; maximum likelihood Q = 48.6807, *I*^2^<0.0001, *p* = 0.8034. For IBD, the MR-Egger results were as follows: Q = 63.1835, *I*^2^ < 0.0001, *p* = 0.8715; IVW *Q* = 65.8841, *I*^2^ < 0.0001, *p* = 0.8343; maximum likelihood *Q* = 65.7086, *I*^2^ < 0.0001, *p* = 0.8382.) (Table 2). The MR-Steiger results supported a causal association between the IBD traits and AS (Table 1). Moreover, we did not detect any outlier SNPs or a horizontal pleiotropic effect of UC, CD, or IBD on the risk of AS when we used the MR-PRESSO global test (*p*-values for UC, CD, and IBD were 0.7020, 0.8060, and 0.7300, respectively). The MR results determined with the outlier-corrected MR-PRESSO method were similar to the MR IVW results reported above (For UC, OR = 1.0256, 95% CI 1.0137–1.0377, *p* = 9.63E-05. For CD, OR = 1.0194, 95% CI 1.0096–1.0293, *p* = 0.0002. For IBD, OR = 1.0238, 95% CI 1.0123–1.0355, *p* = 0.0001) (Table 1). Therefore, the MR-PRESSO results suggested there are causal effects of UC, CD, and IBD on AS.

**TABLE 2:** Heterogeneity and pleiotropy analysis of ulcerative colitis, Crohn’s disease and IBD with ankylosing spondylitis risk using different analytic methods.

Exposure traits	MR methods	Ankylosing spondylitis			
		Cochran *Q* statistic	*I*^2^	Heterogeneity *p-*value	MR-Egger
					Intercept *p-*value
Ulcerative colitis	MR-Egger	43.1297	< 0.0001	0.7434	0.1326
	Inverse variance weighted	45.4670	< 0.0001	0.6923	
	Maximum likelihood	45.2706	< 0.0001	0.6996	
Crohn’s disease	MR-Egger	47.4296	< 0.0001	0.8129	0.2484
	Inverse variance weighted	48.7894	< 0.0001	0.8002	
	Maximum likelihood	48.6807	< 0.0001	0.8034	
IBD	MR-Egger	63.1835	< 0.0001	0.8715	0.1044
	Inverse variance weighted	65.8841	< 0.0001	0.8343	
	Maximum likelihood	65.7086	< 0.0001	0.8382	

*MR, Mendelian randomization; IBD, inflammatory bowel disease.*

**FIGURE 2:**
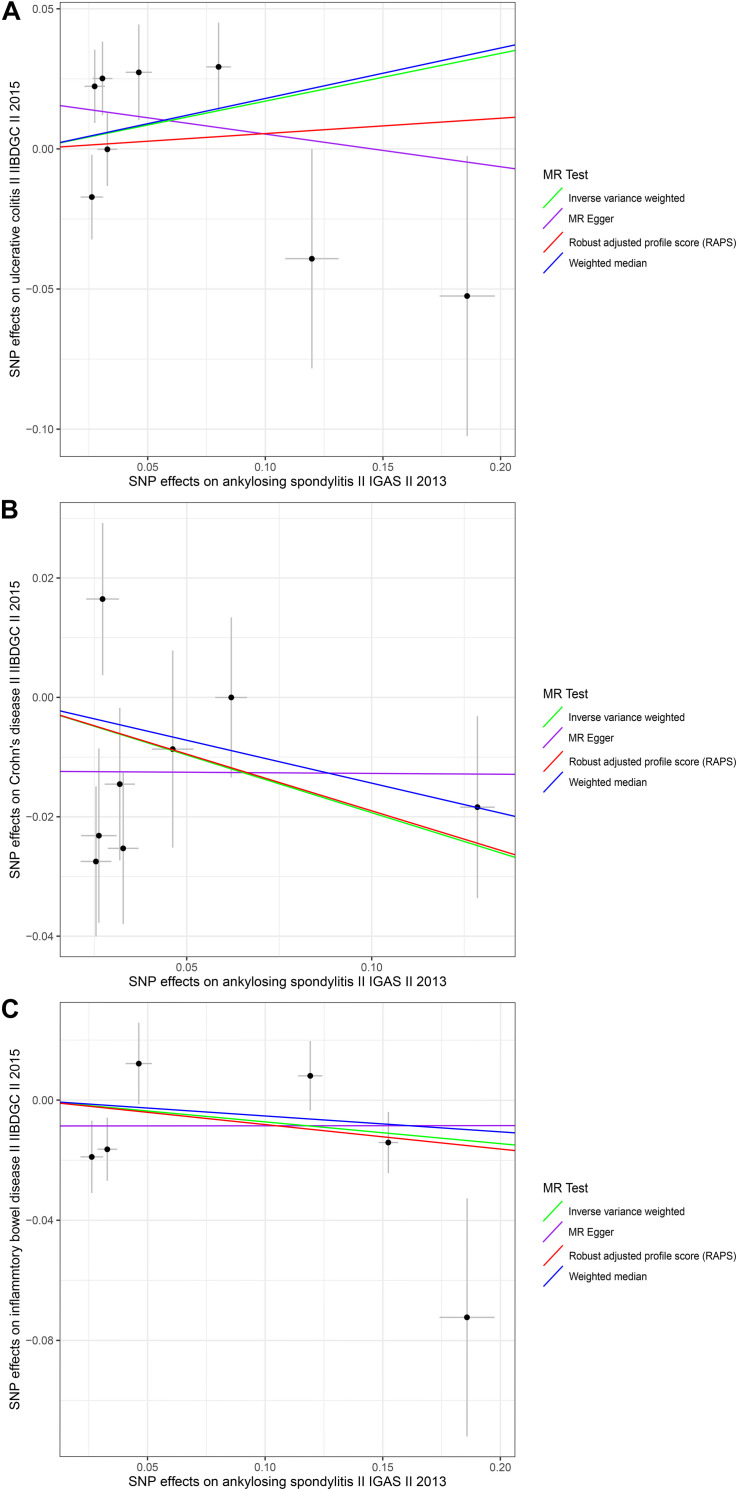
Scatter plots of the genetic associations with ankylosing spondylitis against inflammatory bowel disease risk using different MR methods. **(A)** Ankylosing spondylitis against ulcerative colitis risk; **(B)** ankylosing spondylitis against Crohn’s disease risk; and **(C)** ankylosing spondylitis against inflammatory bowel disease risk. The slopes of each line represent the causal association for each method. The green line represents the inverse variance weighted estimate, the purple line represents the MR-Egger estimate, the red line represents the MR.RAPS estimate, and the blue line represents the weighted median estimate.

To explore the causal effects of AS on UC, CD, and IBD, we incorporated 8, 8, and 6 significant and independent IV SNPs for AS, respectively, which were retrieved from IGAS (Supplementary Tables S7–S9). Overall, the selected instruments explain approximately 0.37, 0.16, and 1.80% of the phenotypic variation of AS on the observed scale. For the instrumental variables of AS, all the *F*-values were greater than 10 (ranging from 30.0213 to 252.1853 with UC; ranging from 30.0213 to 748.6627 with CD and ranging from 30.0213 to 1314.9257 with IBD) with average *F*-values of 105.6131, 156.4408, and 375.1283 for UC, CD, and IBD, respectively (Supplementary Tables S7–S9). There was no evidence suggesting causal associations of an increased risk of AS with changes in the risk of UC, CD, or IBD, based on the IVW, WM, and MR-Egger regression methods and the Bonferroni-corrected significance threshold of *p* < 0.0167 (e.g., 0.05/3) (Supplementary Table S10 and Figure 2). The MR.RAPS and MR-PRESSO test results were consistent with the IVW, WM, and MR-Egger regression results (Supplementary Table S10 and Figure 2). We conducted MR-Egger regression to assess pleiotropy, and the results revealed that the presence of horizontal pleiotropy was unlikely to bias the causality of AS with UC (*p* = 0.2931), CD (*p* = 0.2895), and IBD (*p* = 0.5554) (Supplementary Table S11). The leave-one-out method demonstrated that the observed links were not driven by SNP (see Supplementary Figures S4–S6). Cochran *Q*-value and the *I*^2^-value also indicated there was no heterogeneity across the IV estimates determined with the IVW, MR-Egger, and maximum likelihood methods (Supplementary Table S11). In summary, we did not find significant causal associations of AS with UC, CD, or IBD.

## Discussion

To the best of our knowledge, our study is the first to illustrate the bidirectional causal relationship between IBD and AS using MR analysis and large-scale GWAS data. Our findings provided evidence that IBD (including UC and CD) had positive causal effects on AS risk but did not suggest that there are causal effects of AS on IBD risk in individuals of European descent. We found that suffering from IBD was the causal factor of an increased risk of AS, which suggests that IBD and AS might share a similar pathogenesis.

Although the exact mechanisms linking IBD and AS are not fully understood, the joint-gut axis hypothesis was proposed to explain the pathogenic link (Brakenhoff et al., 2010). Various environmental (gut bacteria-dysbiosis) factors and host factors (migration of activated gut-T cells and macrophages) lead to inflammation in genetically susceptible individuals, which may act as triggers of inflammatory responses against gut and joint components (Brakenhoff et al., 2010; Fragoulis et al., 2019). In one study Tito et al. (2016), investigated the association between intestinal microbiota and spondyloarthritis and demonstrated a significant difference in the intestinal microbial composition between patients with spondyloarthritis who had microscopic gut inflammation and those without microscopic gut inflammation. This study indicated that gut bacteria-dysbiosis might play an important role in the pathogenesis of both diseases. Genetic factors also seem to have a significant impact on linking the two diseases. Laukens et al. (2005) reported that CARD15 gene polymorphisms are associated with an increased risk for chronic gut inflammation in patients with SpA. Peeters et al. (2004) included 102 patients with CD in a study and found that CARD15 variants are genetic predictors of CD-related sacroilitis.

Many studies have shown that the risk of IBD is high in patients with AS (Stolwijk et al., 2014, 2015), but those results did not indicate there are causal effects of AS on the risk of IBD. The findings of some studies were consistent with our findings. With the data from a large population-based public health database in Spain, Muñoz-Ortego et al. (2014) found no significant associations between AS and IBD. In a preliminary cohort study conducted using data from the 2005–2012 database of the Taiwan National Health Insurance Programme in Taiwan, the overall incidence of IBD was lower in the AS group than in the non-AS group, but the difference did not reach statistical significance (Lai et al., 2019). Because there are confounding factors in observational studies, it is unclear whether they are etiologically relevant to each other. The results of our study can provide new information on the similarity of the pathogeneses of the two diseases.

The causal effects of IBD on AS are of great significance for the classification of SpA and the diagnosis and treatment of AS. Traditionally, SpA can be classified as axial SpA or as peripheral SpA. Axial SpA is subclassified as radiographic SpA and non-radiographic SpA based on the presence or absence of definite sacroilitis according to the modified New York Classification Criteria (van der Linden et al., 1984; Rudwaleit et al., 2009). According to the ASAS classification criteria for axial SpA, patients with > 3 months of back pain and age of onset of < 45 years confirmed sacroilitis on imaging examinations, and more than one SpA features (including IBD) or those with HLA-B27 combined with more than two SpA features (including IBD) can be diagnosed with axial SpA. Since we found that IBD is the cause of AS, we recommend that the significance of IBD is emphasized in the axial SpA classification criteria. We also recommend that the features of IBD are included in the modified New York Classification Criteria (van der Linden et al., 1984) for the diagnosis of AS. Our study is also an important addition to IBD and AS research, and the results have important implications for public health. We will predict the occurrence of AS in IBD patients and will provide strategies for preventing and treating AS in IBD patients. For example, surveillance examinations for IBD patients should include not only a regular colonoscopy but also a regular spine X-ray. We also suggest that IBD patients take measures to prevent back injuries that may result in spinal fractures, especially those who have low back pain, because patients with AS are at high risk of fractures (Muñoz-Ortego et al., 2014).

The present study has several limitations. First, the summary-level statistics approach does not allow us to perform analyses stratified by covariates that were adjusted by the original GWAS. Second, we only assumed a linear effect relationship between IBD and AS in the MR model. The summary statistics also did not permit us to explore the non-linearity of the association between IBD and AS. Although linearity is a first-order approximation of any -linear relationship, a simple linearity assumption may not always be reasonable in practice (Burgess et al., 2014). Third, we did not stratify the causal effects between IBD and AS by gender or age, although previous studies revealed that causal effects between IBD and AS can be age and gender dependent (Van Praet et al., 2013). It is difficult to obtain individual-level data in original GWAS. The study population included in the exposure and outcome analyses were of European ancestry, which may have mitigated population stratification. However, the conclusions made based on the European study population are not representative of individuals of other ancestries, such as Asians and Americans. Fourth, the small variance for the exposures, especially for AS with the SNP instruments, might affect the power of the causal effects. The variance might be affected by the small amounts of SNP instruments. However, based on the very large sample size and strongly relevant instruments, we still have been powered to rule in or rule out the causal relationship.

A further limitation is our use of binary risk factors (IBD). IBD is a dichotomization of a continuous risk factor which can lead to violation of the exclusion restriction assumption and limit the inferences drawn from an MR study. In particular, the effect estimate of IBD (yes/no) on AS represents the average effect among individuals for whom the presence or absence of the included genetic effects determines their IBD status. We further assume that the effect of IBD on AS is constant for all individuals, which may not be the case. However, it is important to note that the MR test for an association between IBD and AS is still valid if the instrumental variable assumptions are satisfied (Burgess and Labrecque, 2018). Additionally, we did not propose a physiological mechanism to explain the causal associations between IBD and AS.

## Data Availability Statement

The original contributions presented in the study are included in the article/Supplementary Material, further inquiries can be directed to the corresponding author.

## Author Contributions

YT, ZC, and GH conceptualized and designed the study. XM provided the “TwoSampleMR” package codes in R language and analyzed the data in the study. ZC drafted the manuscript. HF and BH gave constructive suggestions when writing the manuscript. All authors have read the manuscript.

## Conflict of Interest

The authors declare that the research was conducted in the absence of any commercial or financial relationships that could be construed as a potential conflict of interest.

## Footnotes

1 https://gwas.mrcieu.ac.uk/

2 http://www.internationalgenome.org/
