# Supplementary material for: Bidirectional Causal Associations Between Inflammatory Bowel Disease and Ankylosing Spondylitis: A Two-Sample Mendelian Randomization Analysis
Source: Front Genet. 2020 Nov 19;11:587876. doi: 10.3389/fgene.2020.587876 (PMC7710797; doi:10.3389/fgene.2020.587876)
Supplement: Supplementary file 1 [file Data_Sheet_1.pdf]

Table S1. Characteristics of the SNPs used in the Mendelian randomization analysis of the effects of ulcerative colitis on ankylosing spondylitis risk.

Table S2. Characteristics of the SNPs used in the Mendelian randomization analysis of the effects of Crohn's disease on ankylosing spondylitis risk.

Table S3. Characteristics of the SNPs used in the Mendelian randomization analysis of the effects of inflammatory bowel disease on ankylosing spondylitis risk.

Table S4. Associations of the SNPs used in the Mendelian randomization analysis of ulcerative colitis and ankylosing spondylitis risk with potential confounding factors.

Table S5. Associations of the SNPs used in the Mendelian randomization analysis of Crohn's disease and ankylosing spondylitis risk with potential confounding factors.

Table S6. Associations of the SNPs used in the Mendelian randomization analysis of inflammatory bowel disease and ankylosing spondylitis risk with potential confounding factors.

Table S7. Characteristics of the SNPs used in the Mendelian randomization analysis of the effects of ankylosing spondylitis on ulcerative colitis risk.

Table S8. Characteristics of the SNPs used in the Mendelian randomization analysis of the effects of ankylosing spondylitis on Crohn's disease risk.

Table S9. Characteristics of the SNPs used in the Mendelian randomization analysis of the effects of ankylosing spondylitis on IBD risk.

Table S10. MR estimates from each method of assessing the causal effects of ankylosing spondylitis on ulcerative disease, Crohn's disease and IBD risk.

Table S11. Heterogeneity and pleiotropy analysis of ankylosing spondylitis with ulcerative disease, Crohn's disease, and IBD risk using different analytic methods.

Figure S1. "Leave-one-out" sensitivity analysis of causal effects of ulcerative colitis on ankylosing spondylitis.

Figure S2. "Leave-one-out" sensitivity analysis of causal effects of Crohn's disease on ankylosing spondylitis.

Figure S3. "Leave-one-out" sensitivity analysis of causal effects of inflammatory bowel disease on ankylosing spondylitis.

Figure S4. "Leave-one-out" sensitivity analysis of causal effects of ankylosing spondylitis on ulcerative colitis.

Figure S5. "Leave-one-out" sensitivity analysis of causal effects of ankylosing spondylitis on Crohn's disease.

Figure S6. "Leave-one-out" sensitivity analysis of causal effects of ankylosing spondylitis on inflammatory bowel disease.

Target SNPs	Chr	Mapped genes	Effect allele*	Other allele*	Association with ulcerative colitis†				Association with ankylosing spondylitis‡		
					β#	SE	*p*-Value	*F*-Statistic	β	SE	*p*-Value
rs10010325	4	TET2; TET2-AS1	A	C	-0.0830	0.0124	2.18E-11	44.8062	-0.0010	0.0043	0.8158
rs10121077	9	INSL6; JAK2	A	G	0.1151	0.0135	1.99E-17	72.1569	0.0062	0.0045	0.1733
rs10460566	2	DNMT3A	A	G	-0.0818	0.0145	1.60E-08	31.9319	-0.0079	0.0047	0.0938
rs1076137	22	ASCC2	G	A	-0.0823	0.0141	5.18E-09	34.1197	-0.0068	0.0048	0.1602
rs1077773	7	KCCAT333	A	G	0.0721	0.0124	5.96E-09	33.8460	0.0037	0.0041	0.3696
rs10784670	12	LOC107984526	G	A	-0.1169	0.0127	4.59E-20	84.1495	0.0041	0.0042	0.3278
rs10799838	1	RNF186; TMC04	C	T	-0.1289	0.0144	2.87E-19	80.5280	0.0035	0.0048	0.4684
rs11041476	11	LSP1	A	G	0.0735	0.0133	2.92E-08	30.7615	0.0027	0.0044	0.5420
rs11083840	19	PTGIR	G	T	0.0691	0.0125	3.41E-08	30.4589	-0.0059	0.0042	0.1547
rs11229555	11	CNTF; GLYAT	T	G	-0.0823	0.0145	1.21E-08	32.4632	-0.0017	0.0047	0.7146
rs11230563	11	CD6; LOC105369326	T	C	-0.0751	0.0134	1.90E-08	31.5916	-0.0035	0.0044	0.4252
rs11301008	3	TRPC6	C	T	0.1271	0.0207	9.02E-10	37.5267	-0.0088	0.0070	0.2058
rs11641184	16	LITAF	A	C	0.0780	0.0125	4.24E-10	38.9988	0.0049	0.0041	0.2321
rs1182188	7	GNA12	C	T	-0.1076	0.0138	5.03E-15	61.2486	-0.0075	0.0046	0.0991
rs1297256	21	LOC101927745	T	C	-0.1011	0.0127	2.10E-15	62.9730	0.0061	0.0042	0.1492
rs13136827	4	KIAA1109	C	T	-0.1118	0.0176	2.35E-10	40.1550	-0.0058	0.0059	0.3258
rs13255292	8	PVT1	T	C	-0.0755	0.0137	3.82E-08	30.2414	-0.0038	0.0045	0.3968
rs13430791	2	THADA	A	G	0.1059	0.0187	1.39E-08	32.1950	0.0024	0.0068	0.7232
rs1405108	2	LOC105373831; LOC100419513	A	C	-0.0820	0.0132	5.86E-10	38.3683	-0.0037	0.0044	0.3999
rs16939788	18	LINC01882	T	C	0.0808	0.0146	3.46E-08	30.4288	0.0074	0.0046	0.1126
rs17552904	7	IKZF1; SPATA48	T	G	0.0830	0.0141	4.25E-09	34.5043	-0.0009	0.0047	0.8473
rs17679361	17	WIPF2	C	T	0.2472	0.0401	6.80E-10	38.0770	0.0126	0.0146	0.3859
rs17694108	19	SLC7A10; CEBPA-DT	A	G	0.0958	0.0139	6.17E-12	47.2751	0.0081	0.0045	0.0724
rs17780256	17	SLC39A11; LOC105371887	C	A	-0.1154	0.0160	6.13E-13	51.8054	-0.0073	0.0053	0.1650
rs1886731	1	HES5; TNFRSF14	C	T	-0.1405	0.0221	2.25E-10	40.4180	-0.0101	0.0041	0.0134
rs2070729	5	IRF1	A	C	-0.0828	0.0126	4.59E-11	43.3453	-0.0071	0.0042	0.0903
rs2274351	10	SUFU; ACTR1A	T	C	0.0711	0.0130	4.90E-08	29.7576	0.0061	0.0042	0.1418
rs2301989	7	SLC26A3	A	G	-0.1410	0.0217	8.55E-11	42.2195	-0.0029	0.0041	0.4791
rs2395022	7	KPNA7	C	A	-0.1839	0.0292	2.88E-10	39.7549	0.0086	0.0106	0.4173
rs2488401	1	DENND1B	T	C	0.0932	0.0153	1.12E-09	37.1082	-0.0030	0.0050	0.5512
rs272882	5	MIR3936HG; SLC22A4	T	G	0.1459	0.0139	6.67E-26	110.7637	0.0021	0.0045	0.6370
rs2816958	1	NR5A2	G	A	0.1808	0.0205	1.14E-18	77.8060	-0.0034	0.0068	0.6188
rs28624	1	ERRFI1	C	A	-0.1373	0.0174	2.76E-15	62.4315	-0.0032	0.0056	0.5677
rs34659678	6	TRAF3IP2; TRAF3IP2-AS1	T	C	0.2099	0.0251	5.95E-17	69.9926	0.0077	0.0094	0.4122
rs4366152	9	TNFSF15	C	T	0.1204	0.0136	7.79E-19	78.5515	0.0013	0.0042	0.7550
rs4728142	7	IRF5; LOC392787	A	G	0.0970	0.0127	1.92E-14	58.6144	0.0059	0.0042	0.1597
rs4743820	9	LINC00484; LOC100507103	T	C	0.0809	0.0138	4.05E-09	34.6004	0.0040	0.0044	0.3636
rs483905	11	MAML2	A	G	0.1289	0.0228	1.57E-08	31.9636	0.0030	0.0045	0.5057
rs4946717	6	PRDM1; RN7SKP211	T	C	-0.0751	0.0126	2.35E-09	35.6582	0.0039	0.0042	0.3544
rs4973341	2	SNRPGP8; LOC100533842	T	C	0.0735	0.0131	2.25E-08	31.2631	0.0009	0.0045	0.8420
rs59418206	10	CUL2	A	G	0.0736	0.0130	1.45E-08	32.1192	0.0003	0.0039	0.9383

rs596217	11	NXPE2	A	G	-0.092	0.0127	2.39E-13	53.6563	-0.0058	0.0041	0.1560
rs6933404	6	BTF3L4P3; LOC102723649	C	T	0.1668	0.0252	3.68E-11	43.8113	-0.0010	0.0052	0.8483
rs7240004	18	CTIF; LOC105372105	G	A	-0.0824	0.0130	2.50E-10	40.0326	-0.0059	0.0042	0.1605
rs7404095	16	PRKCB	C	T	0.0718	0.0127	1.52E-08	32.0223	0.0068	0.0042	0.1003
rs76546301	7	RNU6-1091P; FIGNL1	A	G	0.2650	0.0410	1.05E-10	41.7277	-0.0190	0.0185	0.3042
rs7752873	6	PRDM1; ATG5	T	C	0.1823	0.0303	1.83E-09	36.1971	0.0098	0.0063	0.1235
rs79045992	16	RPL35AP33; LOC105371321	A	G	0.1181	0.0208	1.43E-08	32.1418	0.0032	0.0068	0.6380
rs941823	13	LINC00598	C	T	0.1087	0.0147	1.39E-13	54.7183	-0.0021	0.0047	0.6554
rs9891119	17	STAT3	C	A	-0.0895	0.0133	1.72E-11	45.2626	-0.0032	0.0042	0.4503
rs9911533	17	CCR7; SMARCE1	T	C	0.0766	0.0132	7.34E-09	33.4439	0.0024	0.0042	0.5666
rs9941524	2	LOC105373831	G	A	0.0977	0.0128	2.15E-14	58.3869	0.0033	0.0041	0.4234

SNP = single nucleotide polymorphism; Chr = chromosome; β = regression effect size; SE= standard error (the standard error is an estimate of the standard deviation (SD) of the coefficient)

*Allele asociated with higher higher risk of ulcerative colitis in European descent.

† Effect size estimates for ulcerative colitis were from International Inflammatory Bowel Disease Genetics Consortium (IIBDGC).

‡ Effect size estimates for ankylosing spondylitis were from International Genetics of Ankylosing Spondylitis Consortium (IGAS).

# Increase in log-odds risk of ulcerative colitis per additional exposure-increasing allele.

Table S1. Characteristics of the SNPs used in the Mendelian randomization analysis of the effects of ulcerative colitis on ankylosing spondylitis risk

Target SNPs	Chr	Mapped genes	Effect allele[*]	Other allele	Association with Crohn's disease[†]				Association with ankylosing spondylitis[‡]		
					β[#]	SE	*p*-Value	*F*-Statistic	β	SE	*p*-Value
rs102275	11	TMEM258	C	T	0.0903	0.0124	3.98E-13	52.6546	0.0021	0.0043	0.6237
rs10276381	7	JAZF1	T	C	0.1159	0.0188	6.75E-10	38.0913	0.0036	0.0061	0.5528
rs11117431	16	LOC105371388; LOC105371389	G	A	-0.1493	0.0164	1.09E-19	82.4366	-0.0082	0.0053	0.1229
rs11648136	16	BRD7; LINC02178	T	C	-0.1266	0.0186	1.06E-11	46.2090	-0.0004	0.0052	0.9386
rs11691685	2	TEX41	G	A	-0.1577	0.0233	1.35E-11	45.7379	-0.0064	0.0084	0.4449
rs12041056	1	IL23R	T	C	0.1084	0.0121	3.51E-19	80.1256	0.0058	0.0042	0.1637
rs12949918	17	STAT3	C	T	-0.1042	0.0124	3.47E-17	71.0556	-0.0070	0.0042	0.0976
rs1297258	21	LOC101927745	T	C	-0.1272	0.0122	2.11E-25	108.4841	0.0064	0.0042	0.1292
rs13204048	6	SLC22A23	C	T	-0.0685	0.0123	2.89E-08	30.7791	-0.0065	0.0042	0.1182
rs16870166	5	LOC105374737	A	G	0.1488	0.0262	1.36E-08	32.2394	0.0036	0.0091	0.6924
rs16917597	10	ZNF365	A	G	-0.2622	0.0460	1.21E-08	32.4772	0.0015	0.0152	0.9216
rs17293632	15	SMAD3	T	C	0.1284	0.0140	3.70E-20	84.5743	-0.0024	0.0050	0.6321
rs17391694	1	LOC107984997; RNFT1P2	T	C	-0.1191	0.0200	2.62E-09	35.4492	-0.0091	0.0064	0.1531
rs17694108	19	SLC7A10; CEBPA-DT	A	G	0.0796	0.0135	3.29E-09	35.0041	0.0081	0.0045	0.0724
rs181826	5	NDFIP1	A	C	0.0996	0.0127	4.53E-15	61.4532	0.0057	0.0043	0.1850
rs1872691	16	ADCY7; BRD7	A	G	-0.1325	0.0158	4.43E-17	70.5743	-0.0095	0.0052	0.0652
rs1990623	16	NKD1; LINC02178	G	A	0.2037	0.0158	4.52E-38	166.4030	-0.0041	0.0061	0.5020
rs2143606	20	OSER1; OSER1-DT	G	A	-0.0742	0.0121	8.36E-10	37.6733	0.0004	0.0045	0.9295
rs2153283	10	IPMK	A	C	-0.1088	0.0155	2.39E-12	49.1310	-0.0026	0.0048	0.5863
rs224140	10	ALDH7A1P4; LOC105378327	T	C	-0.1325	0.0237	2.20E-08	31.3136	-0.0136	0.0075	0.0703
rs2284553	21	IFNGR2	G	A	0.1032	0.0123	5.63E-17	70.1034	-0.0036	0.0041	0.3864
rs2395022	7	KPNA7	C	A	-0.1772	0.0282	3.13E-10	39.5888	0.0086	0.0106	0.4173
rs2488401	1	DENND1B	T	C	0.1205	0.0147	2.07E-16	67.5305	-0.0030	0.0050	0.5512
rs2538470	7	RPL32P17; LOC392145	G	A	-0.0750	0.0123	1.05E-09	37.2371	-0.0047	0.0044	0.2823
rs2945412	17	KSR1	A	G	0.1130	0.0123	4.51E-20	84.1845	-0.0046	0.0042	0.2761
rs303429	10	MAP3K8; CCND3P1	T	C	0.0763	0.0124	8.38E-10	37.6707	0.0005	0.0045	0.9110
rs34592089	4	BANK1	A	G	0.1994	0.0250	1.41E-15	63.7524	0.0063	0.0088	0.4747
rs34779708	10	CREM	G	T	0.1345	0.0124	1.90E-27	117.8204	0.0005	0.0040	0.9007
rs34804116	5	LOC105379031; LOC340090	A	C	-0.0939	0.0127	1.27E-13	54.8958	-0.0033	0.0043	0.4383
rs3812609	9	NOTCH1	T	C	-0.0938	0.0169	2.95E-08	30.7373	-0.0027	0.0057	0.6356
rs3853824	17	C17orf67	C	T	0.0814	0.0126	1.17E-10	41.5093	0.0045	0.0044	0.3052
rs41295117	10	RPL32P23; RBM17	C	T	-0.1129	0.0174	8.90E-11	42.0502	-0.0056	0.0056	0.3171
rs4767956	12	SLC2A13	G	A	-0.0770	0.0123	4.29E-10	38.9782	0.0035	0.0044	0.4242
rs559928	11	RPS6KA4; LINC02723	C	T	0.0991	0.0158	3.75E-10	39.2411	0.0062	0.0053	0.2374
rs56163845	5	CPEB4	G	A	-0.0919	0.0135	9.40E-12	46.4500	-0.0048	0.0047	0.3047
rs568617	11	FIBP	T	C	0.0851	0.0151	1.75E-08	31.7550	0.0075	0.0050	0.1348
rs6074022	20	RPL13P2; CD40	T	C	-0.0963	0.0138	2.70E-12	48.8979	-0.0035	0.0047	0.4544
rs61959448	13	AKAP11; FABP3P2	A	G	0.1073	0.0167	1.50E-10	41.0287	0.0068	0.0058	0.2423
rs6561151	13	LOC107984576	A	G	0.1471	0.0142	4.68E-25	106.9028	0.0070	0.0049	0.1572
rs6679677	1	PHTF1	A	C	-0.1852	0.0221	4.67E-17	70.4724	-0.0091	0.0076	0.2263
rs6738490	2	ATG16L1	C	T	0.2262	0.0121	4.26E-78	349.9869	0.0074	0.0042	0.0737

rs6738825	2	PLCL1	G	A	-0.0748	0.0119	2.86E-10	39.7655	0.0002	0.0036	0.9559
rs6740462	2	LOC105374780	A	C	0.0997	0.0141	1.74E-12	49.7615	0.0078	0.0047	0.0985
rs6827756	4	KIAA1109	C	T	-0.0790	0.0126	3.27E-10	39.5065	-0.0061	0.0042	0.1515
rs7097656	10	TSPAN14	C	T	0.1321	0.0152	3.89E-18	75.3778	0.0008	0.0056	0.8868
rs714027	22	HORMAD2	G	A	-0.0895	0.0123	3.51E-13	52.8998	-0.0028	0.0042	0.5041
rs7194886	16	NOD2; SNX20	T	C	-0.2270	0.0122	1.42E-77	347.5901	-0.0002	0.0038	0.9576
rs7195296	16	SNN; LITAF	C	A	-0.0921	0.0138	2.56E-11	44.4863	0.0071	0.0047	0.1298
rs72727394	15	RASGRP1	T	C	0.1032	0.0150	5.28E-12	47.5795	0.0024	0.0052	0.6473
rs7438704	4	SLAIN2	G	A	0.0839	0.0127	3.42E-11	43.9207	0.0044	0.0042	0.2961
rs752508	19	SBNO2; LOC102725180; LOC102723798	A	G	0.1066	0.0137	7.95E-15	60.3475	-0.0065	0.0044	0.1410
rs76606004	9	LOC645266; TNFSF8	T	C	0.3027	0.0520	5.79E-09	33.9038	0.0002	0.0153	0.9896
rs7758080	6	TAB2; TAB2-AS1	G	A	0.0761	0.0132	7.27E-09	33.4601	-0.0004	0.0051	0.9371
rs77981966	2	THADA	T	C	0.1827	0.0222	2.19E-16	67.4216	0.0063	0.0084	0.4533
rs7848647	9	TNFSF15	C	T	0.1413	0.0130	1.55E-27	118.2245	0.0007	0.0042	0.8661
rs925255	2	FLJ31356; FOSL2	T	C	-0.1000	0.0121	1.07E-16	68.8317	-0.0035	0.0042	0.4074
rs9457247	6	LOC105378120; MIR3939	T	C	0.1237	0.0124	2.08E-23	99.3850	0.0008	0.0044	0.8556
rs9554587	13	CCR12P; UBAC2	G	A	-0.0952	0.0147	8.29E-11	42.1882	-0.0001	0.0046	0.9827
rs974801	4	TET2	G	A	-0.0700	0.0123	1.27E-08	32.3789	-0.0005	0.0043	0.9074

SNP = single nucleotide polymorphism; Chr = chromosome; β = regression effect size; SE= standard error (the standard error is an estimate of the standard deviation (SD) of the coefficient)

*Allele asociated with higher risk of Crohn's disease in European descent.

† Effect size estimates for Crohn's disease were from International Inflammatory Bowel Disease Genetics Consortium (IIBDGC).

‡ Effect size estimates for ankylosing spondylitis were from International Genetics of Ankylosing Spondylitis Consortium (IGAS).

# Increase in log-odds risk of Crohn's disease per additional exposure-increasing allele.

Table S2. Characteristics of the SNPs used in the Mendelian randomization analysis of the effects of Crohn's disease on ankylosing spondylitis

Target SNPs	Chr	Mapped genes	Effect allele*	Other allele*	Association with IBD†				Association with ankylosing spondylitis‡		
					β	SE	*p*-Value	*F*-Statistic	β	SE	*p*-Value
rs10761652	10	ZNF365; LOC105378327	A	G	0.1144	0.0115	2.40E-23	99.1022	0.0047	0.0049	0.3329
rs10878434	12	LRRK2; LOC105369736	A	G	0.0762	0.0118	1.07E-10	41.6818	0.0063	0.0050	0.2075
rs10982431	9	LOC645266; TNFSF8	A	G	0.1341	0.015	6.89E-19	78.7960	-0.0022	0.0063	0.7246
rs11229555	11	CNTF; GLYAT	T	G	-0.0788	0.0114	5.23E-12	47.6005	-0.0017	0.0047	0.7146
rs11230563	11	CD6; LOC105369326	T	C	-0.0811	0.0106	1.71E-14	58.8447	-0.0035	0.0044	0.4252
rs11301008	3	TRPC6	C	T	0.0922	0.0168	4.21E-08	30.0495	-0.0088	0.0070	0.2058
rs11641184	16	LITAF	A	C	0.0773	0.01005	1.09E-14	59.7308	0.0049	0.0041	0.2321
rs11691685	2	TEX41	G	A	-0.1224	0.0188	7.27E-11	42.4452	-0.0064	0.0084	0.4449
rs1182188	7	GNA12	C	T	-0.0659	0.0108	1.08E-09	37.1765	-0.0075	0.0046	0.0991
rs12184386	10	CUL2	T	C	-0.0761	0.0102	8.61E-14	55.6608	0.0012	0.0043	0.7791
rs12585310	13	FGFR10P2P1; RPS21P8	A	G	0.0706	0.0108	5.25E-11	43.0815	-0.0011	0.0042	0.7938
rs1297263	21	LOC101927745	C	A	-0.1134	0.0101	2.10E-29	126.7592	0.0069	0.0042	0.1028
rs13107612	4	BANK1	T	C	0.0733	0.0109	1.62E-11	45.3838	0.0006	0.0044	0.8909
rs13154564	5	LINC02202	C	T	-0.2202	0.0400	3.65E-08	30.3280	-0.0184	0.0164	0.2631
rs13196377	6	TRAF3IP2; TRAF3IP2-AS1	A	G	0.1664	0.0198	4.30E-17	70.6355	0.0087	0.0091	0.3427
rs1363907	5	ERAP1; ERAP2	A	G	0.0815	0.0104	4.87E-15	61.3138	0.0080	0.0042	0.0605
rs1528602	16	LINC02127; LINC02187	C	T	-0.0624	0.0111	2.19E-08	31.3168	0.0052	0.0047	0.2682
rs1535	11	FADS2	G	A	0.0619	0.0104	2.78E-09	35.3289	0.0021	0.0043	0.6288
rs17651741	15	LOC107984739	A	G	0.0702	0.0127	2.81E-08	30.8353	0.0025	0.0055	0.6480
rs17679361	17	WIPF2	C	T	0.1917	0.0330	6.18E-09	33.7773	0.0126	0.0146	0.3859
rs17694108	19	SLC7A10; CEBPA-DT	A	G	0.0858	0.0111	1.21E-14	59.5200	0.0081	0.0045	0.0724
rs17780256	17	SLC39A11; LOC105371887	C	A	-0.0834	0.0126	3.19E-11	44.0546	-0.0073	0.0053	0.1650
rs181826	5	NDFIP1	A	C	0.0820	0.0104	4.05E-15	61.6746	0.0057	0.0043	0.1850
rs1861487	12	LOC107984526	T	C	0.0599	0.0103	5.56E-09	33.9842	-0.0013	0.0042	0.7591
rs1872691	16	ADCY7; BRD7	A	G	-0.1104	0.0130	1.63E-17	72.5412	-0.0095	0.0052	0.0652
rs2050392	10	CCND3P1; NIFKP1	A	G	0.0691	0.0103	1.87E-11	45.0995	0.0001	0.0033	0.9761
rs2108225	7	PIGCP2; SLC26A3	A	G	-0.0675	0.0100	1.27E-11	45.8538	-0.0029	0.0041	0.4791
rs2143606	20	OSER1; OSER1-DT	G	A	-0.0566	0.0100	1.38E-08	32.2109	0.0004	0.0045	0.9295
rs2153283	10	IPMK	A	C	-0.0860	0.012	1.54E-11	45.4879	-0.0026	0.0048	0.5863
rs2274351	10	SUFU; ACTR1A	T	C	0.0605	0.0104	6.93E-09	33.5545	0.0061	0.0042	0.1418
rs2284553	21	IFNGR2	G	A	0.0595	0.0102	4.82E-09	34.2590	-0.0036	0.0041	0.3864
rs2395022	7	KPNA7	C	A	-0.1816	0.0234	8.27E-15	60.2705	0.0086	0.0107	0.4173
rs2488401	1	DENND1B	T	C	0.1071	0.0122	1.43E-18	77.3550	-0.0030	0.0050	0.5512
rs2538470	7	RPL32P17; LOC392145	G	A	-0.0676	0.0102	3.00E-11	44.1771	-0.0047	0.0044	0.2823
rs272882	5	MIR3936HG; SLC22A4	T	G	0.1661	0.0109	1.47E-52	232.7940	0.0021	0.0045	0.6370
rs2769267	1	GBAT2; RFX5; SELENBP1	A	G	0.0680	0.0160	4.10E-09	34.5748	-0.0024	0.0048	0.6207
rs2816958	1	NR5A2	G	A	0.0990	0.0158	3.64E-10	39.2962	-0.0034	0.0068	0.6188

rs2945412	17	KSR1	A	G	0.0563	0.0101	2.77E-08	30.8624	-0.0046	0.0042	0.2761
rs34804116	5	LOC105379031; LOC340090	A	C	-0.0575	0.0104	3.62E-08	30.3407	-0.0033	0.0043	0.4383
rs34856868	1	BTBD8	A	G	-0.1953	0.0341	9.80E-09	32.8808	0.0150	0.0127	0.2397
rs3853824	17	C17orf67	C	T	0.0640	0.0104	7.70E-10	37.8345	0.0045	0.0044	0.3052
rs4692386	4	LOC645481; LOC105374541	C	T	0.0580	0.0102	1.21E-08	32.4676	0.0063	0.0043	0.1398
rs4743820	9	LINC00484; LOC100507103	T	C	0.0640	0.0109	3.80E-09	34.7252	0.0040	0.0044	0.3636
rs4823077	22	HORMAD2	G	A	0.0796	0.0102	4.43E-15	61.4967	0.0028	0.0042	0.5062
rs4845604	1	RORC	A	G	-0.1236	0.0145	1.21E-17	73.1410	0.0096	0.0060	0.1119
rs4946717	6	PRDM1; RN7SKP211	T	C	-0.0965	0.0100	4.37E-22	93.3541	0.0039	0.0042	0.3544
rs4973341	2	SNRPGP8; LOC100533842	T	C	0.0662	0.0104	2.03E-10	40.43920	0.0009	0.0045	0.8420
rs55693740	1	IL12RB2; LOC100130497	A	G	0.1373	0.0204	1.54E-11	45.4876	-0.0064	0.0086	0.4560
rs559928	11	RPS6KA4; LINC02723	C	T	0.0944	0.0130	3.33E-13	53.0056	0.0062	0.0053	0.2374
rs56054640	5	LOC105374736; LOC105374737	C	T	-0.1200	0.0191	3.24E-10	39.5252	-0.0025	0.0073	0.7299
rs56062135	15	SMAD3	T	C	0.1509	0.0198	2.64E-14	58.0829	-0.0036	0.0052	0.4892
rs56163845	5	CPEB4	G	A	-0.0649	0.0111	4.71E-09	34.3040	-0.0048	0.0047	0.3047
rs6074022	20	RPL13P2; CD40	T	C	-0.0743	0.0114	8.32E-11	42.1807	-0.0035	0.0047	0.4544
rs61959439	13	AKAP11; FABP3P2	T	C	0.0905	0.0139	8.15E-11	42.2223	0.0069	0.0059	0.2411
rs6500315	16	LINC02178; NKD1	G	A	0.0766	0.0119	1.12E-10	41.5986	0.0062	0.0052	0.2317
rs6505402	17	CCL2	A	G	-0.0883	0.0105	3.45E-17	71.0698	-0.0053	0.0043	0.2115
rs6561151	13	LOC107984576	A	G	0.1000	0.0119	3.53E-17	71.0205	0.0070	0.0049	0.1572
rs6588248	1	IL23R	G	T	0.0820	0.0099	1.38E-16	68.3274	0.0064	0.0041	0.1192
rs6740462	2	LOC105374780	A	C	0.0800	0.0116	5.59E-12	47.4679	0.0078	0.0047	0.0985
rs67643815	18	CD226	T	G	-0.0629	0.0102	6.42E-10	38.1900	-0.0012	0.0041	0.7679
rs7011507	8	LOC100287157; IDI1P2	A	G	-0.0846	0.0151	2.03E-08	31.4634	-0.0052	0.0060	0.3844
rs7097656	10	TSPAN14	C	T	0.0993	0.0124	1.27E-15	63.9650	0.0008	0.0056	0.8868
rs720325	6	FGFR10P, CCR6	T	G	0.0791	0.0101	3.82E-15	61.7907	0.0018	0.0041	0.6605
rs7240004	18	CTIF; LOC105372105	G	A	-0.0665	0.0103	1.01E-10	41.7936	-0.0059	0.0042	0.1605
rs72978783	6	LINC02539; WAKMAR2	T	C	-0.1504	0.0255	3.68E-09	34.7883	-0.0166	0.0105	0.1125
rs7404095	16	PRKCB	C	T	0.0557	0.0101	3.10E-08	30.6441	0.0068	0.0042	0.1003
rs744166	17	STAT3	G	A	-0.1207	0.0172	2.16E-12	49.2469	-0.0059	0.0042	0.1623
rs7545687	1	TNFRSF9	A	G	-0.1113	0.0143	7.20E-15	60.5426	-0.0072	0.0056	0.1999
rs76177630	10	ZNF365	A	G	0.2153	0.0343	3.62E-10	39.3061	0.0104	0.0122	0.3935
rs77059113	2	THADA	G	T	0.1506	0.0189	1.39E-15	63.7826	0.0079	0.0085	0.3573
rs7773324	6	IRF4, DUSP22	A	G	0.0618	0.0106	5.84E-09	33.8880	0.0016	0.0042	0.7045
rs7969592	12	IFNG; LOC105369818	G	A	-0.0817	0.0099	1.41E-16	68.2870	-0.0045	0.0042	0.2837
rs8055876	16	CLEC16A	A	G	0.1023	0.0161	2.02E-10	40.4445	-0.0069	0.0064	0.2765
rs925255	2	FLJ31356; FOSL2	T	C	-0.0759	0.0100	2.70E-14	57.9397	-0.0035	0.0042	0.4074
rs928722	6	LOC102723649; LOC105378018	T	C	0.0956	0.0123	6.46E-15	60.7558	-0.0010	0.0052	0.8483
rs941823	13	LINC00598	C	T	0.0830	0.0115	6.19E-13	51.7867	-0.0021	0.0047	0.6554
rs9486285	6	PRDM1; ATG5	T	C	0.0856	0.0135	2.26E-10	40.2288	0.0057	0.0058	0.3298
rs9557207	13	UBAC2	G	A	-0.0878	0.0121	3.52E-13	52.8966	1.00E-04	0.0051	0.9844
rs974801	4	TET2	G	A	-0.0728	0.0101	7.07E-13	51.5234	-0.0005	0.0043	0.9074

SNP = single nucleotide polymorphism; Chr = chromosome; IBD = inflammatory bowel disease; β = regression effect size; SE = standard error (the standard error is an estimate of the standard deviation (SD) of the coefficient)

*Allele asociated with higher risk of IBD in European descent.

† Effect size estimates for ulcerative colitis were from International Inflammatory Bowel Disease Genetics Consortium (IIBDGC).

‡ Effect size estimates for ankylosing spondylitis were from International Genetics of Ankylosing Spondylitis Consortium (IGAS).

# Increase in log-odds risk of IBD per additional exposure-increasing allele.

Table S3. Characteristics of the SNPs used in the Mendelian randomization analysis of the effects of inflammatory bowel disease on ankylosing spondylitis risk.

Exposure	SNP	Chr	Mapped genes	Effect allele*	Confounding factors	*p*-Value	Sample size (N)
Ulcerative colitis	rs10010325	4	TET2; TET2-AS1	A	osteoarthritis	0.0828	50508
Ulcerative colitis	rs10121077	9	INSL6; JAK2	A	osteoarthritis	0.0992	50508
Ulcerative colitis	rs10460566	2	DNMT3A	A	osteoarthritis	0.2057	50508
Ulcerative colitis	rs1076137	22	ASCC2	G	osteoarthritis	0.0133	50508
Ulcerative colitis	rs1077773	7	KCCAT333	A	osteoarthritis	0.7141	50508
Ulcerative colitis	rs10784670	12	LOC107984526	G	osteoarthritis	0.3239	50508
Ulcerative colitis	rs10799838	1	RNF186; TMC04	C	osteoarthritis	0.7814	50508
Ulcerative colitis	rs11041476	11	LSP1	A	osteoarthritis	0.1312	50508
Ulcerative colitis	rs11083840	19	PTGIR	G	osteoarthritis	0.5356	50508
Ulcerative colitis	rs11229555	11	CNTF; GLYAT	T	osteoarthritis	0.1480	50508
Ulcerative colitis	rs11230563	11	CD6; LOC105369326	T	osteoarthritis	0.5315	50508
Ulcerative colitis	rs11301008	3	TRPC6	C	osteoarthritis	0.1163	50508
Ulcerative colitis	rs11641184	16	LITAF	A	osteoarthritis	0.0821	50508
Ulcerative colitis	rs1182188	7	GNA12	C	osteoarthritis	0.4992	50508
Ulcerative colitis	rs1297256	21	LOC101927745	T	osteoarthritis	0.3751	50508
Ulcerative colitis	rs13136827	4	KIAA1109	C	osteoarthritis	0.2935	50508
Ulcerative colitis	rs13255292	8	PVT1	T	osteoarthritis	0.7731	50508
Ulcerative colitis	rs13430791	2	THADA	A	osteoarthritis	0.1489	50508
Ulcerative colitis	rs1405108	2	LOC105373831; LOC100419513	A	osteoarthritis	0.2930	50508
Ulcerative colitis	rs16939788	18	LINC01882	T	osteoarthritis	0.5278	50508
Ulcerative colitis	rs17552904	7	IKZF1; SPATA48	T	osteoarthritis	0.3302	50508
Ulcerative colitis	rs17679361	17	WIPF2	C	osteoarthritis	0.0413	50508
Ulcerative colitis	rs17694108	19	SLC7A10; CEBPA-DT	A	osteoarthritis	0.5721	50508
Ulcerative colitis	rs17780256	17	SLC39A11; LOC105371887	C	osteoarthritis	0.0392	50508
Ulcerative colitis	rs1886731	1	HES5; TNFRSF14	C	osteoarthritis	0.5751	50508
Ulcerative colitis	rs2070729	5	IRF1	A	osteoarthritis	0.5478	50508
Ulcerative colitis	rs2274351	10	SUFU; ACTR1A	T	osteoarthritis	0.8781	50508
Ulcerative colitis	rs2301989	7	SLC26A3	A	osteoarthritis	0.0038	50508
Ulcerative colitis	rs2395022	7	KPNA7	C	osteoarthritis	0.9564	50508
Ulcerative colitis	rs2488401	1	DENND1B	T	osteoarthritis	0.4311	50508
Ulcerative colitis	rs272882	5	MIR3936HG; SLC22A4	T	osteoarthritis	0.0566	50508
Ulcerative colitis	rs2816958	1	NR5A2	G	osteoarthritis	0.0325	50508
Ulcerative colitis	rs28624	1	ERRFI1	C	osteoarthritis	0.8461	50508
Ulcerative colitis	rs34659678	6	TRAF3IP2; TRAF3IP2-AS1	T	osteoarthritis	0.0031	50508
Ulcerative colitis	rs4366152	9	TNFSF15	C	osteoarthritis	0.4629	50508
Ulcerative colitis	rs4728142	7	IRF5; LOC392787	A	osteoarthritis	0.8997	50508
Ulcerative colitis	rs4743820	9	LINC00484; LOC100507103	T	osteoarthritis	0.5471	50508
Ulcerative colitis	rs483905	11	MAML2	A	osteoarthritis	0.0425	50508
Ulcerative colitis	rs4946717	6	PRDM1; RN7SKP211	T	osteoarthritis	0.5124	50508
Ulcerative colitis	rs4973341	2	SNRPGP8; LOC100533842	T	osteoarthritis	0.7699	50508
Ulcerative colitis	rs59418206	10	CUL2	A	osteoarthritis	0.0511	50508
Ulcerative colitis	rs596217	11	NXPE2	A	osteoarthritis	0.9001	50508

Ulcerative colitis	rs6933404	6	BTF3L4P3; LOC102723649	C	osteoarthritis	0.9077	50508
Ulcerative colitis	rs7240004	18	CTIF; LOC105372105	G	osteoarthritis	0.5551	50508
Ulcerative colitis	rs7404095	16	PRKCB	C	osteoarthritis	0.9334	50508
Ulcerative colitis	rs76546301	7	RNU6-1091P; FIGNL1	A	osteoarthritis	0.6525	50508
Ulcerative colitis	rs7752873	6	PRDM1; ATG5	T	osteoarthritis	0.6925	50508
Ulcerative colitis	rs79045992	16	RPL35AP33; LOC105371321	A	osteoarthritis	0.6770	50508
Ulcerative colitis	rs941823	13	LINC00598	C	osteoarthritis	0.0971	50508
Ulcerative colitis	rs9891119	17	STAT3	C	osteoarthritis	0.8840	50508
Ulcerative colitis	rs9911533	17	CCR7; SMARCE1	T	osteoarthritis	0.5694	50508
Ulcerative colitis	rs9941524	2	LOC105373831	G	osteoarthritis	0.3604	50508

SNP = single nucleotide polymorphism; Chr = chromosome; *Allele asociated with higher risk of ulcerative colitis.

Table S4. Associations of the SNPs used in the Mendelian randomization analysis of ulcerative colitis and ankylosing spondylitis risk with potential confounding factors.

Exposure	SNP	Chr	Mapped genes	Effect allele*	Confounding factors	*p*-Value	Sample size (N)
Crohn’s disease	rs102275	11	TMEM258	C	osteoarthritis	0.7428	50508
Crohn’s disease	rs10276381	7	JAZF1	T	osteoarthritis	0.5437	50508
Crohn’s disease	rs11117431	16	LOC105371388; LOC105371389	G	osteoarthritis	0.4446	50508
Crohn’s disease	rs11648136	16	BRD7; LINC02178	T	osteoarthritis	0.0980	50508
Crohn’s disease	rs11691685	2	TEX41	G	osteoarthritis	0.6922	50508
Crohn’s disease	rs12041056	1	IL23R	T	osteoarthritis	0.7243	50508
Crohn’s disease	rs12949918	17	STAT3	C	osteoarthritis	0.7976	50508
Crohn’s disease	rs1297258	21	LOC101927745	T	osteoarthritis	0.4471	50508
Crohn’s disease	rs13204048	6	SLC22A23	C	osteoarthritis	0.5501	50508
Crohn’s disease	rs16870166	5	LOC105374737	A	osteoarthritis	0.2457	50508
Crohn’s disease	rs16917597	10	ZNF365	A	osteoarthritis	0.6631	50508
Crohn’s disease	rs17293632	15	SMAD3	T	osteoarthritis	0.0858	50508
Crohn’s disease	rs17391694	1	LOC107984997; RNFT1P2	T	osteoarthritis	0.0636	50508
Crohn’s disease	rs17694108	19	SLC7A10; CEBPA-DT	A	osteoarthritis	0.5721	50508
Crohn’s disease	rs181826	5	NDFIP1	A	osteoarthritis	0.6094	50508
Crohn’s disease	rs1872691	16	ADCY7; BRD7	A	osteoarthritis	0.5324	50508
Crohn’s disease	rs1990623	16	NKD1; LINC02178	G	osteoarthritis	0.8664	50508
Crohn’s disease	rs2143606	20	OSER1; OSER1-DT	G	osteoarthritis	0.1879	50508
Crohn’s disease	rs2153283	10	IPMK	A	osteoarthritis	0.1346	50508
Crohn’s disease	rs224140	10	ALDH7A1P4; LOC105378327	T	osteoarthritis	0.8529	50508
Crohn’s disease	rs2284553	21	IFNGR2	G	osteoarthritis	0.2528	50508
Crohn’s disease	rs2395022	7	KPNA7	C	osteoarthritis	0.9564	50508
Crohn’s disease	rs2488401	1	DENND1B	T	osteoarthritis	0.4311	50508
Crohn’s disease	rs2538470	7	RPL32P17; LOC392145	G	osteoarthritis	0.1300	50508
Crohn’s disease	rs2945412	17	KSR1	A	osteoarthritis	0.4928	50508
Crohn’s disease	rs303429	10	MAP3K8; CCND3P1	T	osteoarthritis	0.8643	50508
Crohn’s disease	rs34592089	4	BANK1	A	osteoarthritis	0.9881	50508
Crohn’s disease	rs34779708	10	CREM	G	osteoarthritis	0.0506	50508
Crohn’s disease	rs34804116	5	LOC105379031; LOC340090	A	osteoarthritis	0.3584	50508
Crohn’s disease	rs3812609	9	NOTCH1	T	osteoarthritis	0.8299	50508
Crohn’s disease	rs3853824	17	C17orf67	C	osteoarthritis	0.1752	50508
Crohn’s disease	rs41295117	10	RPL32P23; RBM17	C	osteoarthritis	0.8794	50508
Crohn’s disease	rs4767956	12	SLC2A13	G	osteoarthritis	0.6294	50508
Crohn’s disease	rs559928	11	RPS6KA4; LINC02723	C	osteoarthritis	0.5059	50508
Crohn’s disease	rs56163845	5	CPEB4	G	osteoarthritis	0.9152	50508
Crohn’s disease	rs568617	11	FIBP	T	osteoarthritis	0.4346	50508
Crohn’s disease	rs6074022	20	RPL13P2; CD40	T	osteoarthritis	0.7568	50508
Crohn’s disease	rs61959448	13	AKAP11; FABP3P2	A	osteoarthritis	0.0532	50508
Crohn’s disease	rs6561151	13	LOC107984576	A	osteoarthritis	0.3792	50508
Crohn’s disease	rs6679677	1	PHTF1	A	osteoarthritis	0.6727	50508
Crohn’s disease	rs6738490	2	ATG16L1	C	osteoarthritis	0.2202	50508
Crohn’s disease	rs6738825	2	PLCL1	G	osteoarthritis	0.0709	50508

Crohn's disease	rs6740462	2	LOC105374780	A	osteoarthritis	0.4337	50508
Crohn's disease	rs6827756	4	KIAA1109	C	osteoarthritis	0.5596	50508
Crohn's disease	rs7097656	10	TSPAN14	C	osteoarthritis	0.0084	50508
Crohn's disease	rs714027	22	HORMAD2	G	osteoarthritis	0.8069	50508
Crohn's disease	rs7194886	16	NOD2; SNX20	T	osteoarthritis	0.0891	50508
Crohn's disease	rs7195296	16	SNN; LITAF	C	osteoarthritis	0.6684	50508
Crohn's disease	rs72727394	15	RASGRP1	T	osteoarthritis	0.4190	50508
Crohn's disease	rs7438704	4	SLAIN2	G	osteoarthritis	0.5811	50508
Crohn's disease	rs752508	19	SBNO2; LOC102725180; LOC102723798	A	osteoarthritis	0.2238	50508
Crohn's disease	rs76606004	9	LOC645266; TNFSF8	T	osteoarthritis	0.3726	50508
Crohn's disease	rs7758080	6	TAB2; TAB2-AS1	G	osteoarthritis	0.5888	50508
Crohn's disease	rs77981966	2	THADA	T	osteoarthritis	0.0420	50508
Crohn's disease	rs7848647	9	TNFSF15	C	osteoarthritis	0.5158	50508
Crohn's disease	rs925255	2	FLJ31356; FOSL2	T	osteoarthritis	0.3083	50508
Crohn's disease	rs9457247	6	LOC105378120; MIR3939	T	osteoarthritis	0.3282	50508
Crohn's disease	rs9554587	13	CCR12P; UBAC2	G	osteoarthritis	0.4551	50508
Crohn's disease	rs974801	4	TET2	G	osteoarthritis	0.0183	50508

SNP = single nucleotide polymorphism; Chr = chromosome; *Allele asociated with higher risk of Crohn's disease.

Table S5. Associations of the SNPs used in the Mendelian randomization analysis of Crohn's disease and ankylosing spondylitis risk with potential confounding factors.

Exposure	SNP	Chr	Mapped genes	Effect allele*	Confounding factors	*p*-Value	Sample size (N)
IBD	rs10761652	10	ZNF365; LOC105378327	A	osteoarthritis	0.8483	50508
IBD	rs10878434	12	LRRK2; LOC105369736	A	osteoarthritis	0.9382	50508
IBD	rs10982431	9	LOC645266; TNFSF8	A	osteoarthritis	0.2174	50508
IBD	rs11229555	11	CNTF; GLYAT	T	osteoarthritis	0.1480	50508
IBD	rs11230563	11	CD6; LOC105369326	T	osteoarthritis	0.5315	50508
IBD	rs11301008	3	TRPC6	C	osteoarthritis	0.1163	50508
IBD	rs11641184	16	LITAF	A	osteoarthritis	0.0821	50508
IBD	rs11691685	2	TEX41	G	osteoarthritis	0.4309	50508
IBD	rs1182188	7	GNA12	C	osteoarthritis	0.6922	50508
IBD	rs12184386	10	CUL2	T	osteoarthritis	0.4992	50508
IBD	rs12585310	13	FGFR10P2P1; RPS21P8	A	osteoarthritis	0.4035	50508
IBD	rs1297263	21	LOC101927745	C	osteoarthritis	0.1319	50508
IBD	rs13107612	4	BANK1	T	osteoarthritis	0.2779	50508
IBD	rs13154564	5	LINC02202	C	osteoarthritis	0.3258	50508
IBD	rs13196377	6	TRAF3IP2; TRAF3IP2-AS1	A	osteoarthritis	0.8894	50508
IBD	rs1363907	5	ERAP1; ERAP2	A	osteoarthritis	0.0018	50508
IBD	rs1528602	16	LINC02127; LINC02187	C	osteoarthritis	0.5174	50508
IBD	rs1535	11	FADS2	G	osteoarthritis	0.3332	50508
IBD	rs17651741	15	LOC107984739	A	osteoarthritis	0.9560	50508
IBD	rs17679361	17	WIPF2	C	osteoarthritis	0.3134	50508
IBD	rs17694108	19	SLC7A10; CEBPA-DT	A	osteoarthritis	0.0413	50508
IBD	rs17780256	17	SLC39A11; LOC105371887	C	osteoarthritis	0.5721	50508
IBD	rs181826	5	NDFIP1	A	osteoarthritis	0.0392	50508
IBD	rs1861487	12	LOC107984526	T	osteoarthritis	0.6094	50508
IBD	rs1872691	16	ADCY7; BRD7	A	osteoarthritis	0.6867	50508
IBD	rs2050392	10	CCND3P1; NIFKP1	A	osteoarthritis	0.5324	50508
IBD	rs2108225	7	PIGCP2; SLC26A3	A	osteoarthritis	0.7736	50508
IBD	rs2143606	20	OSER1; OSER1-DT	G	osteoarthritis	0.0132	50508
IBD	rs2153283	10	IPMK	A	osteoarthritis	0.1879	50508
IBD	rs2274351	10	SUFU; ACTR1A	T	osteoarthritis	0.1346	50508
IBD	rs2284553	21	IFNGR2	G	osteoarthritis	0.8781	50508
IBD	rs2395022	7	KPNA7	C	osteoarthritis	0.2528	50508
IBD	rs2488401	1	DENND1B	T	osteoarthritis	0.956	50508
IBD	rs2538470	7	RPL32P17; LOC392145	G	osteoarthritis	0.4311	50508
IBD	rs272882	5	MIR3936HG; SLC22A4	T	osteoarthritis	0.1300	50508
IBD	rs2769267	1	GBAT2; RFX5; SELENBP1	A	osteoarthritis	0.0991	50508
IBD	rs2816958	1	NR5A2	G	osteoarthritis	0.0566	50508
IBD	rs2945412	17	KSR1	A	osteoarthritis	0.5224	50508
IBD	rs34804116	5	LOC105379031; LOC340090	A	osteoarthritis	0.0325	50508
IBD	rs34856868	1	BTBD8	A	osteoarthritis	0.4928	50508
IBD	rs3853824	17	C17orf67	C	osteoarthritis	0.9726	50508

IBD	rs4692386	4	LOC645481; LOC105374541	C	osteoarthritis	0.3584	50508
IBD	rs4743820	9	LINC00484; LOC100507103	T	osteoarthritis	0.3266	50508
IBD	rs4823077	22	HORMAD2	G	osteoarthritis	0.1752	50508
IBD	rs4845604	1	RORC	A	osteoarthritis	0.5323	50508
IBD	rs4946717	6	PRDM1; RN7SKP211	T	osteoarthritis	0.5471	50508
IBD	rs4973341	2	SNRPGP8; LOC100533842	T	osteoarthritis	0.6895	50508
IBD	rs55693740	1	IL12RB2; LOC100130497	A	osteoarthritis	0.1694	50508
IBD	rs559928	11	RPS6KA4; LINC02723	C	osteoarthritis	0.5124	50508
IBD	rs56054640	5	LOC105374736; LOC105374737	C	osteoarthritis	0.7699	50508
IBD	rs56062135	15	SMAD3	T	osteoarthritis	0.8399	50508
IBD	rs56163845	5	CPEB4	G	osteoarthritis	0.5059	50508
IBD	rs6074022	20	RPL13P2; CD40	T	osteoarthritis	0.1812	50508
IBD	rs61959439	13	AKAP11; FABP3P2	T	osteoarthritis	0.0852	50508
IBD	rs6500315	16	LINC02178; NKD1	G	osteoarthritis	0.9152	50508
IBD	rs6505402	17	CCL2	A	osteoarthritis	0.7568	50508
IBD	rs6561151	13	LOC107984576	A	osteoarthritis	0.0610	50508
IBD	rs6588248	1	IL23R	G	osteoarthritis	0.8740	50508
IBD	rs6740462	2	LOC105374780	A	osteoarthritis	0.5805	50508
IBD	rs67643815	18	CD226	T	osteoarthritis	0.3792	50508
IBD	rs7011507	8	LOC100287157; IDI1P2	A	osteoarthritis	0.8862	50508
IBD	rs7097656	10	TSPAN14	C	osteoarthritis	0.4337	50508
IBD	rs720325	6	FGFR10P, CCR6	T	osteoarthritis	0.2222	50508
IBD	rs7240004	18	CTIF; LOC105372105	G	osteoarthritis	0.0952	50508
IBD	rs72978783	6	LINC02539; WAKMAR2	T	osteoarthritis	0.0084	50508
IBD	rs7404095	16	PRKCB	C	osteoarthritis	0.7817	50508
IBD	rs744166	17	STAT3	G	osteoarthritis	0.5551	50508
IBD	rs7545687	1	TNFRSF9	A	osteoarthritis	0.0543	50508
IBD	rs76177630	10	ZNF365	A	osteoarthritis	0.9334	50508
IBD	rs77059113	2	THADA	G	osteoarthritis	0.7988	50508
IBD	rs7773324	6	IRF4, DUSP22	A	osteoarthritis	0.9301	50508
IBD	rs7969592	12	IFNG; LOC105369818	G	osteoarthritis	0.4686	50508
IBD	rs8055876	16	CLEC16A	A	osteoarthritis	0.0672	50508
IBD	rs925255	2	FLJ31356; FOSL2	T	osteoarthritis	0.0780	50508
IBD	rs928722	6	LOC102723649; LOC105378018	T	osteoarthritis	0.9491	50508
IBD	rs941823	13	LINC00598	C	osteoarthritis	0.1129	50508
IBD	rs9486285	6	PRDM1; ATG5	T	osteoarthritis	0.3083	50508
IBD	rs9557207	13	UBAC2	G	osteoarthritis	0.9440	50508
IBD	rs974801	4	TET2	G	osteoarthritis	0.0971	50508

SNP = single nucleotide polymorphism; Chr = chromosome; IBD = inflammatory bowel disease; *Allele asociated with higher risk of inflammatory bowel disease.

Table S6. Associations of the SNPs used in the Mendelian randomization analysis of inflammatory bowel disease and ankylosing spondylitis risk with potential confounding factors.

Target SNPs	Chr	Mapped genes	Effect allele*	Other allele*	Association with ankylosing spondylitis†				Association with ulcerative colitis‡		
					β	SE	*p*-Value	*F*-Statistic	β	SE	*p*-Value
rs11065898	12	SH2B3	T	C	0.0263	0.0048	4.71E-08	30.0213	-0.0172	0.0151	0.2552
rs17336532	6	ZBED9	T	C	-0.0801	0.0053	1.47E-52	228.4090	-0.0293	0.0158	0.0639
rs1860545	12	TNFRSF1A	A	G	-0.0275	0.0044	2.78E-10	39.0625	-0.0223	0.0131	0.0869
rs2074475	6	TRIM15	C	T	0.1858	0.0117	1.09E-56	252.1853	-0.0525	0.0500	0.2938
rs4129267	1	IL6R	T	C	-0.0308	0.0042	3.32E-13	53.7778	-0.0251	0.0131	0.0555
rs6600247	1	RUNX3; LOC105376878	C	T	0.0328	0.0042	2.58E-15	60.98873	-0.0001	0.0131	0.9920
rs7771335	6	MAS1LP1; LOC105375008	G	A	-0.1198	0.0113	4.27E-26	112.3975	0.0392	0.0391	0.3169
rs9393646	6	CARMIL1	A	G	0.0462	0.0056	1.10E-16	68.0625	0.0273	0.0171	0.1089

SNP = single nucleotide polymorphism; Chr = chromosome; β = regression effect size; SE= standard error (the standard error is an estimate of the standard deviation (SD) of the coefficient)

*Allele asociated with higher risk of ulcerative colitis in European descent.

† Effect size estimates for ankylosing spondylitis were from International Genetics of Ankylosing Spondylitis Consortium (IGAS).

‡ Effect size estimates for ulcerative disease were from International Inflammatory Bowel Disease Genetics Consortium (IIBDGC).

# Increase in log-odds risk of IBD per additional exposure-increasing allele.

Table S7. Characteristics of the SNPs used in the Mendelian randomization analysis of the effects of ankylosing spondylitis on ulcerative colitis risk.

Target SNPs	Chr	Mapped genes	Effect allele*	Other allele*	Association with ankylosing spondylitis†				Association with Crohn's disease‡		
					β	SE	*p*-Value	*F*-Statistic	β	SE	*p*-Value
rs11065898	12	SH2B3	T	C	0.0263	0.0048	4.71E-08	30.0213	-0.0232	0.01460	0.1126
rs12615545	2	LINC01934	C	T	0.0255	0.0042	1.03E-09	36.8622	-0.0275	0.0126	0.0287
rs130067	6	CCHCR1	G	T	0.1286	0.0047	8.61E-166	748.6627	-0.0184	0.0152	0.2275
rs27529	5	ERAP1	G	A	-0.0620	0.0043	3.28E-47	207.8962	1.16E-05	0.0134	0.9993
rs2927613	5	ERAP1	A	G	-0.0273	0.0044	7.07E-10	38.4964	-0.0165	0.0128	0.1964
rs6600247	1	RUNX3; LOC105376878	C	T	0.0328	0.0042	2.58E-15	60.9887	-0.0253	0.0127	0.0460
rs9393646	6	CARMIL1	A	G	0.0462	0.0056	1.10E-16	68.0625	-0.0087	0.0165	0.5995
rs9901869	17	MRPL45P2; NPEPPS	A	G	0.0319	0.0041	6.04E-15	60.5360	-0.0145	0.0128	0.2554

SNP = single nucleotide polymorphism; Chr = chromosome; β = regression effect size; SE= standard error (the standard error is an estimate of the standard deviation (SD) of the coefficient)

*Allele asociated with higher risk of Crohn's disease in European descent.

† Effect size estimates for ankylosing spondylitis were from International Genetics of Ankylosing Spondylitis Consortium (IGAS).

‡ Effect size estimates for Crohn's disease were from International Inflammatory Bowel Disease Genetics Consortium (IIBDGC).

# Increase in log-odds risk of IBD per additional exposure-increasing allele.

Table S8. Characteristics of the SNPs used in the Mendelian randomization analysis of the effects of ankylosing spondylitis on Crohn's disease risk.

Target SNPs	Chr	Mapped genes	Effect allele*	Other allele*	Association with ankylosing spondylitis†				Association with IBD‡		
					β	SE	*p*-Value	*F*-Statistic	β	SE	*p*-Value
rs11065898	12	SH2B3	T	C	0.0263	0.0048	4.71E-08	30.0213	-0.0189	0.0120	0.1163
rs2074475	6	TRIM15	C	T	0.1858	0.0117	1.09E-56	252.1853	-0.0723	0.0396	0.0682
rs2272593	6	PRRC2A; MIR6832	C	T	0.1191	0.0052	1.72E-117	524.5862	0.0081	0.0116	0.4830
rs2596501	6	HLA-B	T	C	-0.1523	0.0042	1.00E-200	1314.9257	0.0141	0.0102	0.1672
rs6600247	1	RUNX3; LOC105376878	C	T	0.0328	0.0042	2.58E-15	60.9887	-0.0163	0.0105	0.1188
rs9393646	6	CARMIL1	A	G	0.0462	0.0056	1.10E-16	68.0625	0.0122	0.0136	0.3691

SNP = single nucleotide polymorphism; Chr = chromosome; IBD = inflammatory bowel disease; β = regression effect size; SE= standard error (the standard error is an estimate of the standard deviation (SD) of the coefficient)

*Allele asociated with higher risk of IBD in European descent.

† Effect size estimates for ankylosing spondylitis were from International Genetics of Ankylosing Spondylitis Consortium (IGAS).

‡ Effect size estimates for ulcerative disease were from International Inflammatory Bowel Disease Genetics Consortium (IIBDGC).

# Increase in log-odds risk of IBD per additional exposure-increasing allele.

Table S9. Characteristics of the SNPs used in the Mendelian randomization analysis of the effects of ankylosing spondylitis on IBD risk.

Exposure traits	MR methods	Ulcerative colitis				Crohn's disease				IBD			
		Number of SNPs	OR (95% CI)	SE	MR *p*-value	Number of SNPs	OR (95% CI)	SE	MR *p*-value	Number of SNPs	OR (95% CI)	SE	MR *p*-value
**Ankylosing spondylitis**	MR-Egger	8	0.8900 (0.4993~1.5866)	0.2949	0.7065	8	0.9961 (0.6770~1.4655)	0.1970	0.9847	6	1.0009 (0.7661~1.3078)	0.1364	0.9948
	Inverse variance weighted	8	1.1858 (0.8639~1.6278)	0.1616	0.2916	8	0.8244 (0.6599~1.0299)	0.1136	0.0890	6	0.9304 (0.8098~1.0689)	0.0708	0.3083
	Weighted median	8	1.1973 (0.8618~1.6633)	0.1677	0.2831	8	0.8662 (0.7050~1.0643)	0.1051	0.1715	6	0.9488 (0.8430~1.0679)	0.0603	0.3839
	Robust adjusted profile score	8	1.0563 (0.7722~1.4449)	0.1598	0.7320	8	0.8269 (0.6549~1.0442)	0.1190	0.1103	6	0.9220 (0.7994~1.0635)	0.0728	0.2651
	MR-PRESSO test	8	1.1858 (0.8639~1.6278)	0.1616	0.3266	8	0.8244 (0.6599~1.0299)	0.1136	0.1328	6	0.9304 (0.8098~1.0689)	0.0708	0.3550

MR = Mendelian randomization; PRESSO = Pleiotropy RESidual Sum and Outlier; SNP = single nucleotide polymorphism; IBD = inflammatory bowel disease;

OR = odds ratio; CI = confidence interval; SE = standard error (the standard error is an estimate of the standard deviation (SD) of the coefficient)

Table S10. MR estimates from each method of assessing the causal effects of ankylosing spondylitis on ulcerative disease, Crohn's disease and IBD risk.

Exposure traits	MR methods	Ulcerative colitis				Crohn’s disease				IBD			
		Cochran *Q* statistic	I^2	Heterogeneity *p*-value	MR-Egger Intercept *p*-value	Cochran *Q* statistic	I^2	Heterogeneity *p*-value	MR-Egger Intercept *p*-value	Cochran *Q* statistic	I^2	Heterogeneity *p*-value	MR-Egger Intercept *p*-value
**Ankylosing spondylitis**	MR-Egger	11.3042	0.3808	0.0794	0.2931	9.2307	0.3500	0.1610	0.2895	8.5803	0.5338	0.0725	0.5554
	Inverse variance weighted	13.8050	0.4205	0.0548		11.3069	0.3809	0.1258		9.4664	0.4718	0.0918	
	Maximum likelihood	13.7678	0.4189	0.0555		11.2604	0.3784	0.1277		9.4589	0.4714	0.0921	

MR = Mendelian randomization; IBD = inflammatory bowel disease

Table S11. Heterogeneity and pleiotropy analysis of ankylosing spondylitis with ulcerative disease, Crohn’s disease, and IBD risk using different analytic methods.

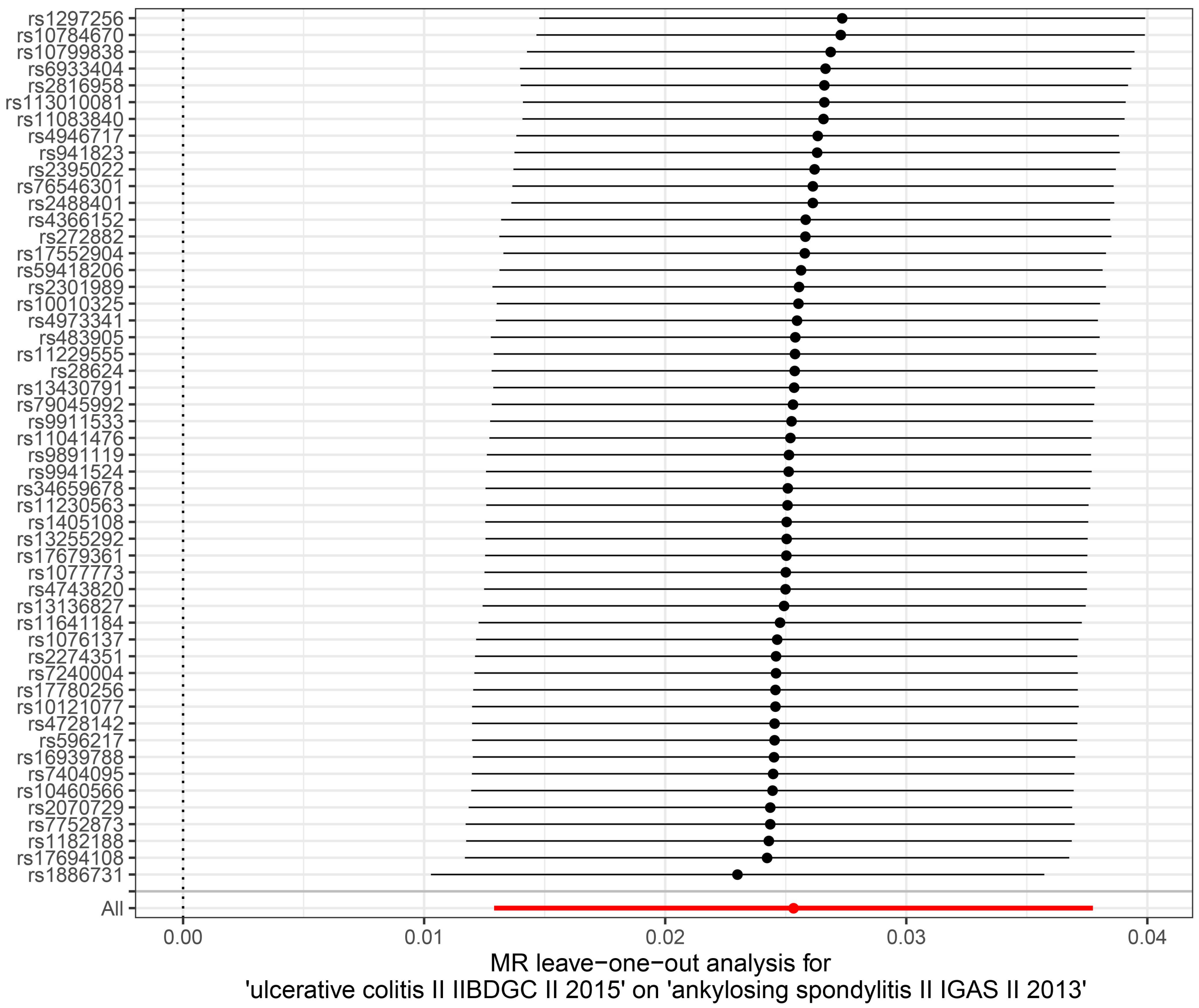

Figure S1. "Leave-one-out" sensitivity analysis of causal effects of ulcerative colitis on ankylosing spondylitis. The significance of the red line is the MR results of the inverse variance weighting (IVW) method.

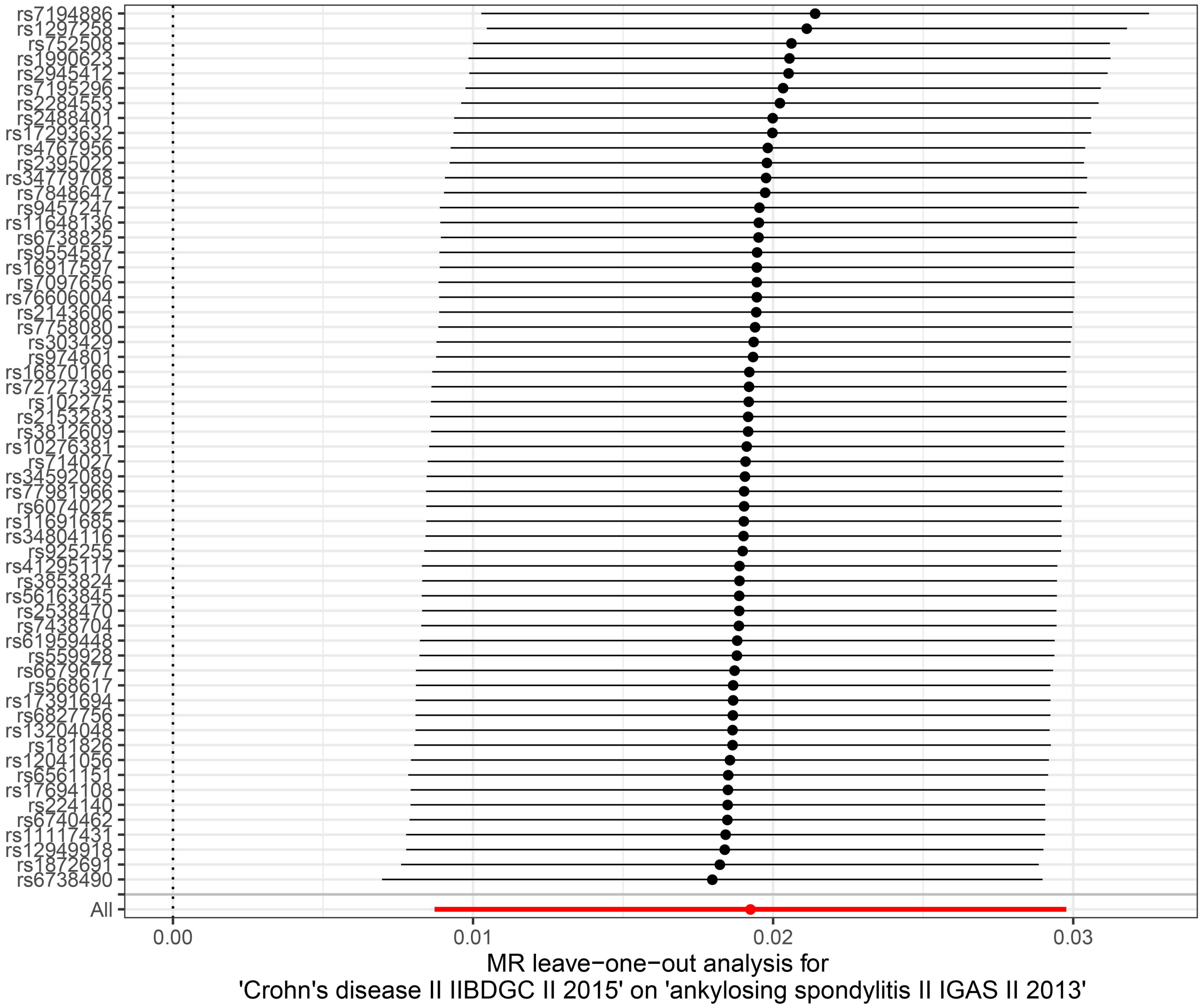

Figure S2. “Leave-one-out” sensitivity analysis of causal effects of Crohn’s disease on ankylosing spondylitis. The significance of the red line is the MR results of the inverse variance weighting (IVW) method.

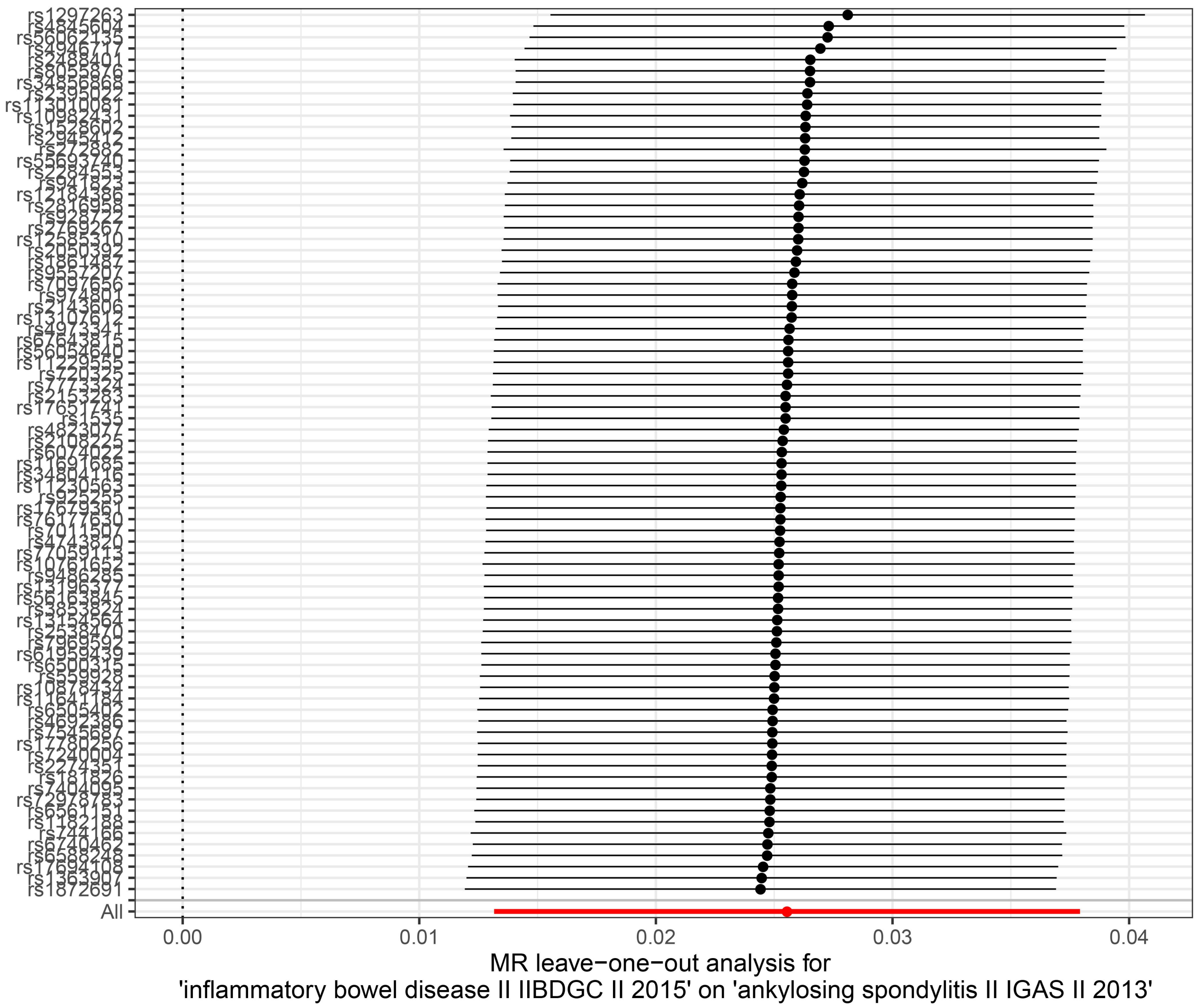

Figure S3. "Leave-one-out" sensitivity analysis of causal effects of inflammatory bowel disease on ankylosing spondylitis. The significance of the red line is the MR results of the inverse variance weighting (IVW) method.

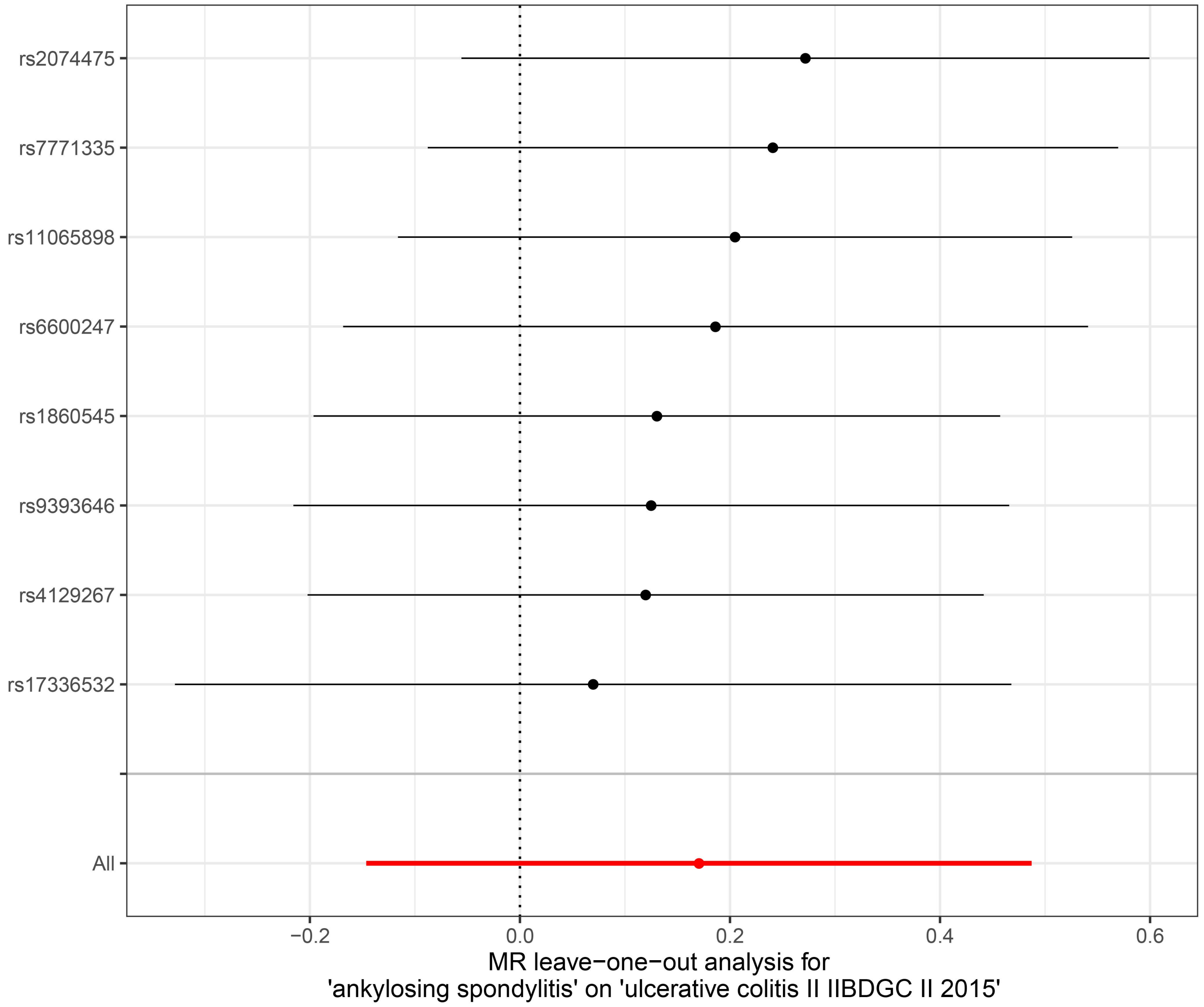

Figure S4. "Leave-one-out" sensitivity analysis of causal effects of ankylosing spondylitis on ulcerative colitis. The significance of the red line is the MR results of the inverse variance weighting (IVW) method.

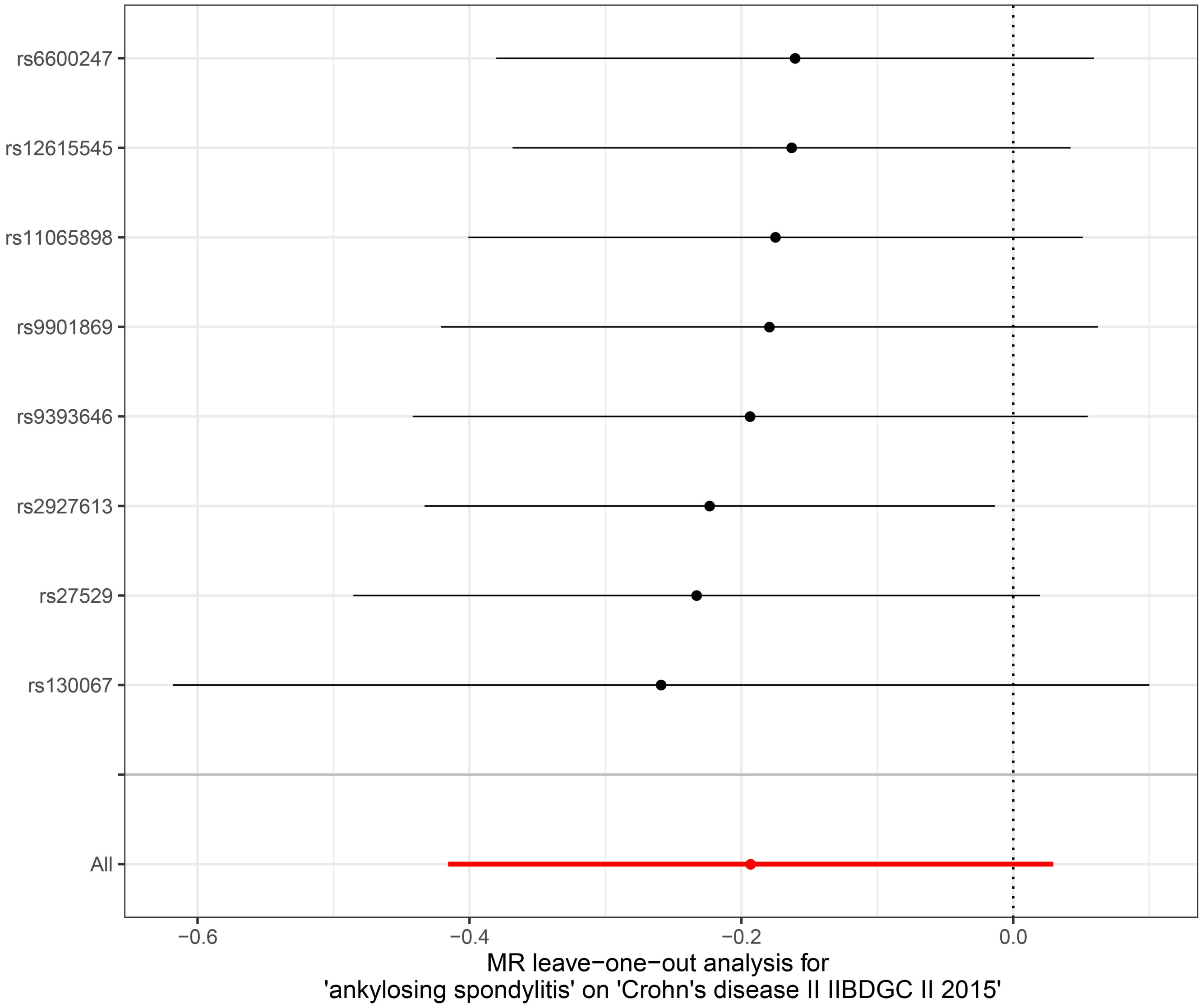

Figure S5. "Leave-one-out" sensitivity analysis of causal effects of ankylosing spondylitis on Crohn's disease. The significance of the red line is the MR results of the inverse variance weighting (IVW) method. Note: The p-value after removing rs2927613 in the MR leave-one-out analysis is 0.0369, which is not statistically significant at a Bonferroni-corrected $p < 0.0167$ (e.g., 0.05/3 outcomes).

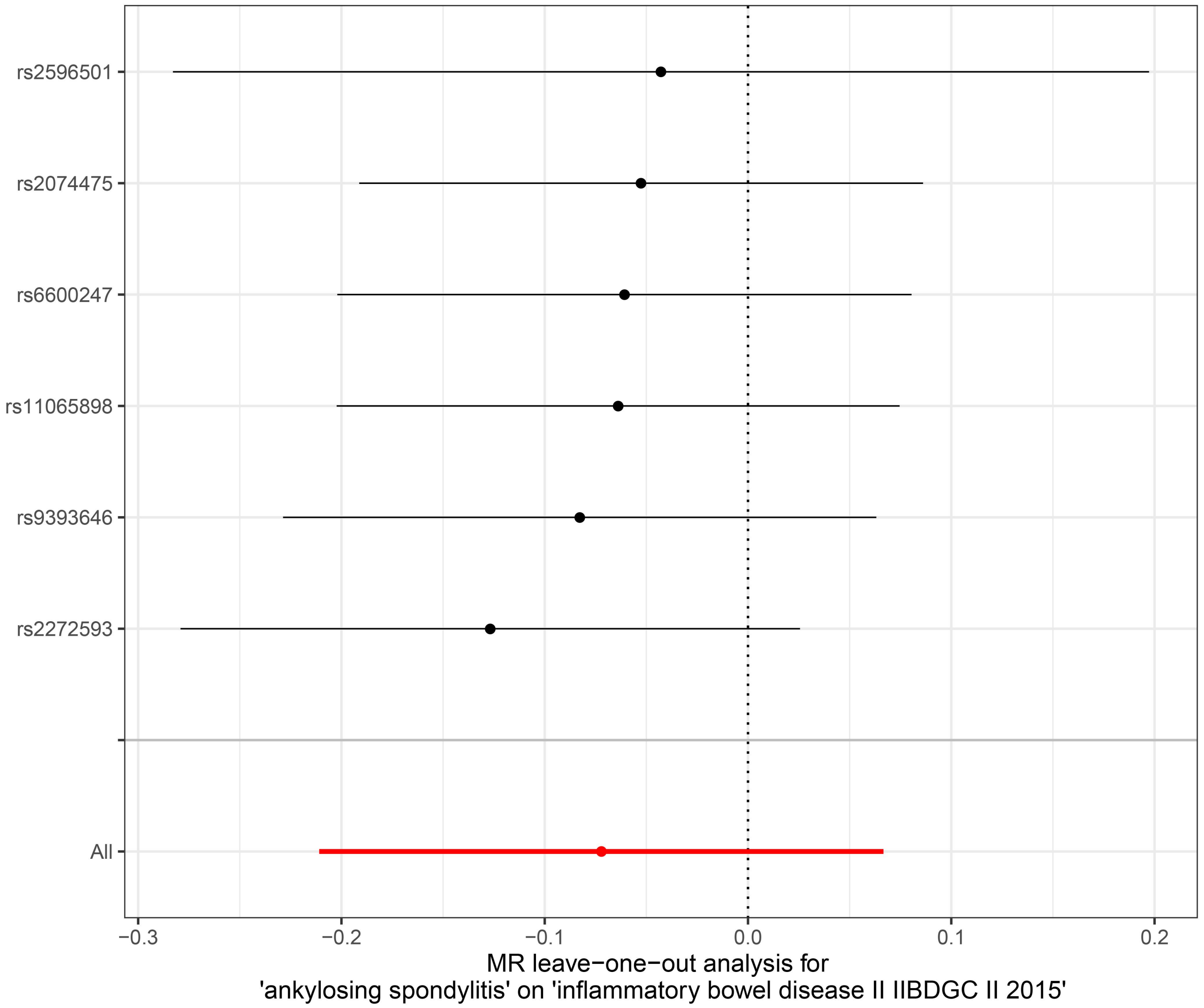

Figure S6. “Leave-one-out” sensitivity analysis of causal effects of ankylosing spondylitis on inflammatory bowel disease. The significance of the red line is the MR results of the inverse variance weighting (IVW) method.
